# Snapshot Spatiotemporal‐Spectral Imaging Enabled by Joint Photonic Encoding

**DOI:** 10.1002/advs.78383

**Published:** 2026-10-22

**Authors:** Chunyu Chen, Junren Wen, Hao Sha, Chenying Yang, Ziyang Chen, Xiaochen Feng, Taiqin Chen, Wenzhen Zou, Xiangyu Chen, Xiangyang Ji, Guojun Liu, Yongbing Zhang

**Affiliations:** ^1^ Faculty of Computing Harbin Institute of Technology Harbin China; ^2^ Hangzhou Institute for Advanced Study University of Chinese Academy of Sciences Hangzhou Zhejiang China; ^3^ School of Computer Science and Technology Harbin Institute of Technology (Shenzhen) Shenzhen China; ^4^ School of Physics Harbin Institute of Technology Harbin Heilongjiang China; ^5^ Department of Automation Tsinghua University Beijing China

**Keywords:** computational reconstruction, dynamic spectral sensing, high‐dimensional optical imaging, spatiotemporal‐spectral photonic encoding

## Abstract

Hyperspectral imaging (HSI) enables simultaneous acquisition of spectral and spatial information and has been widely adopted across science and engineering. However, existing HSI systems are primarily designed for x‐y‐λ datacube acquisition, with the temporal dimension insufficiently incorporated and limiting their ability to capture rapid variations in spatial structures or spectral signatures. Here we report Spectra4D (x, y, λ, t), a snapshot spatiotemporal‐spectral imaging architecture that enables high‐dimensional and high‐resolution observation of dynamic scenes beyond the native camera frame‐rate limit. Enabled by joint photonic encoding approach, Spectra4D synergizes high‐speed temporal modulation with on‐sensor spectral multiplexing to jointly encode spatiotemporal and spectral information into a single compressed exposure, which is computationally reconstructed using a dual‐mask guided reconstruction network. Experiments demonstrate that Spectra4D reconstructs 28 visible spectral channels under 4× temporal compression, achieving spectral fidelity above 96.5% and pixel‐level localization accuracy across continuous multi‐frame sequences. We further validate the capability of Spectra4D in dynamic spatio‐spectral scenarios, including dual‐color fluorescent microsphere tracking and frame‐resolved elemental monitoring in flame combustion. In summary, Spectra4D presents a practical and scalable route toward temporally resolved spatio‐spectral imaging of dynamic processes with rapidly varying spectral features.

## Graphical abstract

Spectra4D enables snapshot spatiotemporal‐spectral imaging by combining high‐speed DMD temporal modulation with CMOS‐integrated Fabry‐Pérot spectral encoding. From a single 2D measurement, a dual‐mask‐guided reconstruction network recovers multiple time‐resolved hyperspectral frames, enabling high‐fidelity observation of rapidly evolving spatial, spectral, and temporal dynamics.

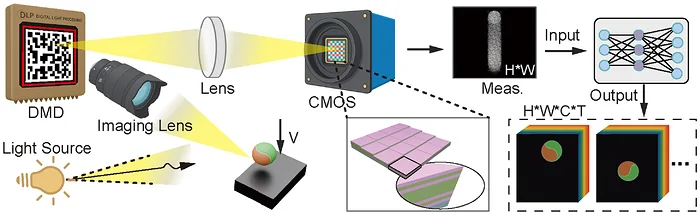

## 1 Introduction

Hyperspectral imaging (HSI) provides spatially resolved spectral information by sampling multiple narrow wavelength channels, enabling direct discrimination of spectral differences beyond conventional intensity‐based imaging [1, 2]. This combination of spatial fidelity and spectral specificity makes HSI a powerful tool for material analysis and chemical characterization.

Although HSI has been widely used in many static scenarios, including medical diagnosis, crop assessment, remote sensing, and biomedical imaging [3, 4, 5, 6, 7], when spectrally informative signatures vary over time, resolving their spatiotemporal dynamics becomes essential. In many practical dynamic settings, such as rapid chemical and bio‐fluorescent reactions [8, 9, 10, 11, 12], both spectral signatures and spatial distribution change concurrently, placing stringent demands on the temporal resolution of hyperspectral imaging systems.

Existing HSI methods mostly rely on scanning or sequential acquisition [13] strategies, thus if dynamic processes occur at frequencies higher than the system will inevitably suffer from motion blur and spectral aliasing. Recent advances have further extended hyperspectral imaging toward higher acquisition speeds through alternative spectral‐encoding mechanisms. For example, AOTF‐based spectral selection enables full‐field spatial acquisition with rapid electronic wavelength tuning, although the spectral dimension is acquired sequentially [14]. Snapshot hyperspectral imaging (SHI) systems address this limitation by encoding spectral information into a single compressed measurement followed by computational reconstruction [15, 16]. More recently, nonlinear spectral encoding has enabled single‐exposure broadband spatial‐spectral imaging through wavelength‐dependent nonlinear optical encoding [17]. Yet SHI architectures still remain constrained by the native camera frame rate [18, 19, 20, 21], and spectral multiplexing inevitably introduces mixing that degrade reconstruction fidelity. Alternative strategies have attempted to recover the spectral information via optical flow interpolation [8] or time‐multiplexed sequential sampling [22, 23]. For example, spatial‐temporal multiplexing combined with dispersive Fourier transformation maps spectral information onto high‐speed temporal waveforms, enabling rapid spectral acquisition at the expense of sequential spatial sampling in single‐pixel implementations [24]. These approaches implicitly assume spectral stability and motion consistency across channels. However, the assumption fails when the target exhibits rapid spectral shifts or coupled spatiotemporal variations. Essentially, HSI methods commonly construct hyperspectral videos by time‐sequentially acquiring a series of x‐y‐λ data cubes, leaving their temporal resolution fundamentally constrained by the readout frequency of data cubes. Overall, achieving high spatiotemporal‐spectral resolution in highly dynamic scenarios remains a significant challenge, arising from multiple factors: (i) hardware‐induced temporal limitations; (ii) information loss under strong compression; and (iii) intrinsic instability of spectral and spatial features during rapid evolution.

The representative approaches discussed above offer different balances among spatial parallelism, spectral acquisition speed, and optical complexity. To address this challenge, we develop Spectra4D, a snapshot spatiotemporal‐spectral imaging architecture for high‐dimensional observation of dynamic x‐y‐λ‐t scenes. Spectra4D is built on integrated photonic encoding [25, 26, 27, 28], which enables spatially registered spectral multiplexing without dispersive mixing and provides a simplified forward model for high‐fidelity reconstruction [29, 30, 31, 32, 33]. Specifically, Spectra4D uses a Fabry–Pérot microcavity array for on‐sensor spectral encoding and combines it with high‐speed temporal modulation using a digital micromirror device (DMD), jointly encoding spatial, spectral, and temporal information within a single exposure. Followed by a dual‐mask‐guided end to end reconstruction network, Spectra4D recovers multi‐frame hyperspectral sequences from a single measurement, successfully surpassing the native camera frame rate while preserving spatial and spectral fidelity (Figure 1a). Through a global inversion strategy, Spectra4D reduces spatial information loss from spectral superpixel grouping or direct interpolation [34, 35], enabling high dimensional datacube reconstruction at fine spatial resolution. Simulations and experiments validate the performance of Spectra4D under a high output‐to‐readout dimensionality ratio of 112:1, spanning 28 spectral channels across 410–680 nm with a temporal ratio of 4:1. In simulation, reconstructed images achieve PSNR (Peak Signal‐to‐Noise Ratio) values above 35 dB. Experimentally, spectral fidelity exceeds 96.5%, spatial resolution reaches 2 lp mm^−1^, and localization error remains within 2 pixels over continuous multi‐frame observations.

**FIGURE 1:**
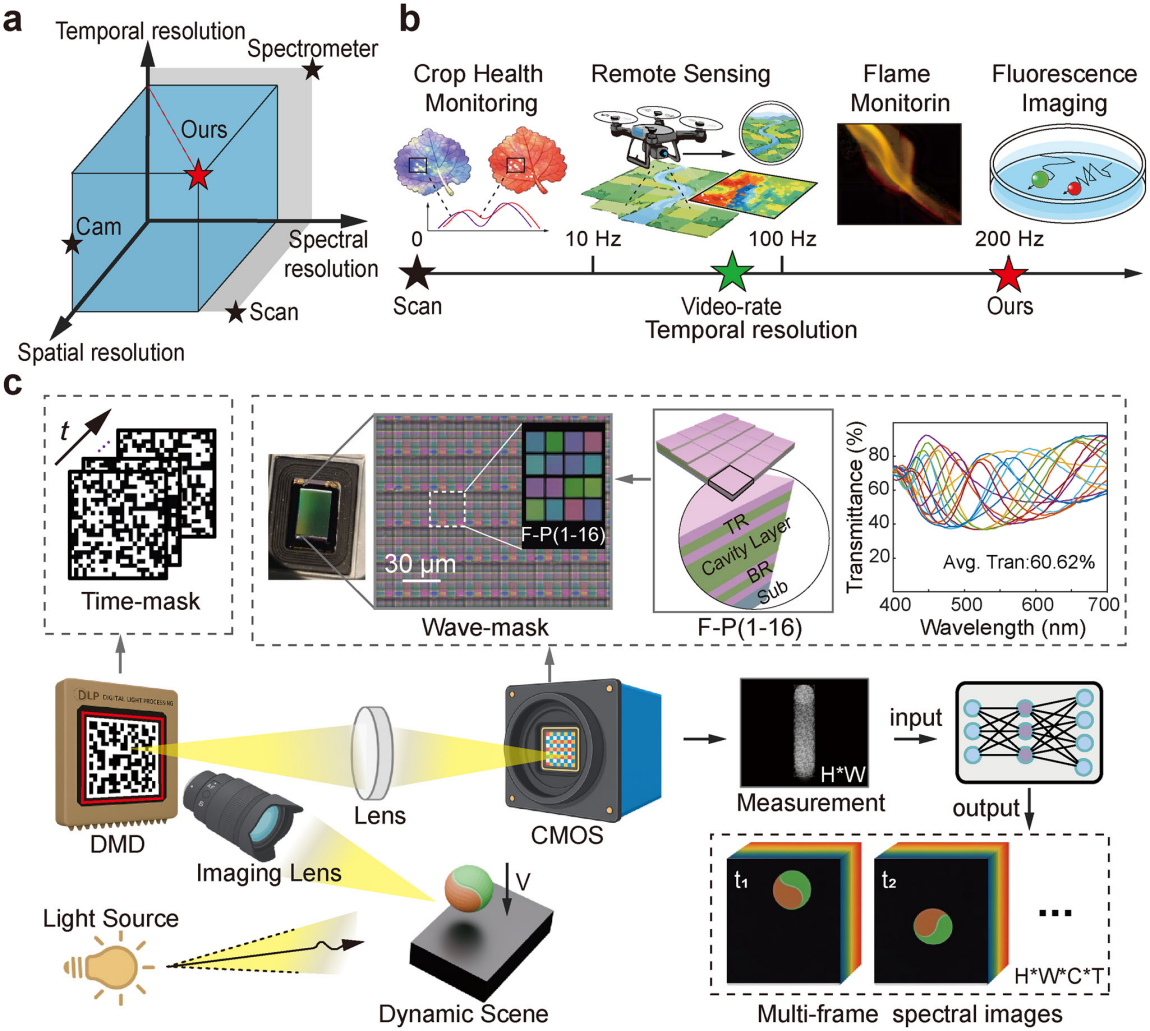
Concept and Schematic of the Spectra4D. a Conceptual 3D trade‐off space spanned by temporal‐spatial‐spectral resolution, illustrating the operating regimes of conventional camera (Cam), point spectrometer (Spectrometer), and scanning‐based hyperspectral acquisition (Scan). The proposed Spectra4D targets a regime that jointly improves temporal sampling while maintaining multi‐band spectral discrimination. b Representative application scenarios and corresponding time resolutions, ranging from low‐speed scanning tasks (e.g., crop health monitoring) through video‐rate scenarios (e.g., remote sensing) to 200 Hz regimes (e.g., fluorescence imaging and flame monitoring), highlighting where Spectra4D extends HSI beyond the native camera frame rate. c System schematic: high‐speed temporal masks are sequentially displayed on a DMD during a single exposure, while an on‐chip Fabry‐Pérot microcavity mosaic periodically tiled with 16 distinct cavity designs (F‐P1–F‐P16) provides spatially varying spectral responses. The 2D measurement is computationally decoded by a reconstruction network to recover a time‐resolved hyperspectral data cube.

Moreover, with simultaneous 4D (x, y, λ and t) sensing capability, Spectra4D enables reliable measurement of spectral dynamics across multiple temporal scales (Figure 1b). For example, in multispectral fluorescent bead tracking, Spectra4D achieves spectral discrimination of distinct bead species without any fluorophore‐specific filter in microscopic system, while supporting fast kinematic analysis. In flame combustion experiments, Spectra4D enables frame‐resolved monitoring of elemental composition dynamics and the corresponding spatial distribution during combustion. These results highlight the ability of Spectra4D to provide reliable quantitative spatiotemporal‐spectral measurements in rapidly evolving and complex conditions, advancing hyperspectral video imaging toward practical dynamic sensing.

## 2 Results

### 2.1 Overview of Spectra4D System

The general outline of Spectra4D is shown in Figure 1c, which integrates on‐chip microfilter‐based spatial‐spectral encoding with DMD‐based temporal modulation for joint temporal‐spectral acquisition. During a single camera exposure, the scene is sequentially modulated by a series of high‐speed temporal patterns generated by the DMD, encoding multiple temporal states into a single sensor measurement. Under this temporal coding scheme, several temporal frames can be recovered from a single 2D readout.

In parallel with the temporal modulation, spatial‐spectral information is encoded using an ultra‐compact on‐chip spectral sensor based on a Fabry‐Pérot (F‐P) micro‐filter array. The F‐P microfilters consist of distributed Bragg reflectors (DBRs) enclosing a cavity layer whose thickness varies spatially across the sensor. The DBRs are formed by alternating high‐ and low‐index dielectric layers to provide broadband reflectivity (Figure S1). According to the resonance condition, the cavity length determines the resonant transmission wavelength, such that spatial variation in cavity thickness produces distinct spectral passbands (Figure S2). This establishes a direct mapping between spatial position and spectral response without relying on dispersive spreading (Figure S3). Such all‐optical encoding scheme eliminates the need for mechanical spectral scanning, making the simultaneous capture of both spatial and spectral information possible. The overall encoded process can be expressed as:

(1) $$y\;\left(u,v\right)=\int_{t_{\min}}^{t_{\max}}\int_{\lambda_{\min}}^{\lambda_{\max}}n\left(u,v,t\right)\;\cdot m\left(u,v,\lambda \right)\cdot x\left(u,v,\lambda ,t\right)\;d\lambda dts$$

where *u*,  *v* denote the spatial coordinates of the image, and *y*(*u*,  *v*) represents the compressed snapshot measurement acquired by the sensor. The term *x*(*u*,  *v*,  λ,  *t*) denotes the original spatio‐spectral‐temporal information. The *m*(*u*,  *v*,  λ) represents the spectral response of the micro‐filter array, and *n*(*u*,  *v*,  *t*) denotes the temporal modulation pattern generated by the DMD.

To simplify the formulation, we discretize the continuous spectrum into *C* spectral channels and consider *T* temporal frames. The dynamic hyperspectral scene can be represented as ${\left\{X_{t,c}\right\}}_{t=1,c=1}^{T,C}\in R^{U\times V}$, where *U* and *V* denote the spatial resolution of each frame, *C* is the number of spectral bands (e.g., *C* = 28), and *T* is the number of temporally coded frames (e.g., *T* = 4). The temporal encoding is implemented via a sequence of binary masks *M_t_* $\in R^{U\times V}$ loaded onto the DMD, and the spectral encoding is realized through the spectral response maps of the micro‐filter array, denoted as ${\left\{F_{c}\right\}}_{c=1}^{C}\in R^{U\times V}$. The final measurement $Y\in R^{U\times V}$ captured by the CMOS sensor can be mathematically modeled as:

(2) $$Y=\sum_{t=1}^{T}\sum_{c=1}^{C}X_{t,c}\;⊙M_{t}⊙F_{c}+N$$

where ⊙ denotes the Hadamard product, and $N\in R^{U\times V}$ represents additive measurement noise. The recovery of the spatiotemporal‐spectral datacube can then be formulated as a physically constrained inverse problem with the spectral and temporal encoding matrices Φ_λ_ and Φ_*t*_:

(3) $$\hat{x}=\arg\min_{x}|Y-\Phi_{t}\Phi_{\lambda }x{|}^{2}+\tau R\left(x\right)$$

where R(x) denotes the regularization term and τ denotes the regularization parameter. Based on the compressed measurement and the calibrated encoding masks, the underlying spatio‐spectral‐temporal datacube can then be computationally reconstructed to yield a time‐resolved hyperspectral video.

### 2.2 Computational Reconstruction and Comparison of Spectra4D System

For computational reconstruction, the temporal modulation and spectral encoding are designed to be independent, allowing stable reconstruction from the compressed measurement. The complete encoding pipeline is summarized in Figure 2a. A physics‐aware reconstruction decodes the multiplexed measurement to recover a time‐resolved sequence of spectral images, yielding spatio‐spectral video imaging beyond the native frame rate of the camera. However, recovering a multi‐frame hyperspectral datacube from a single snapshot is fundamentally ill‐posed under such extreme compression. We therefore develop a dual‐mask‐guided reconstruction network (DM‐NET, Figure S4 and Note S1) that embeds the known temporal and spectral encoding as physical constraints to reduce the reconstruction artifacts and recover time‐resolved hyperspectral videos. Inspired by efficientSCI‐NET [36, 37], DM‐NET adopts a hybrid CNN‐transformer architecture to model long‐range correlations across spatiotemporal and spectral dimensions. Additionally, the network utilizes Mean Squared Error (MSE) loss to refine the reconstruction accuracy by minimizing the error between the predicted and ground truth data.

**FIGURE 2:**
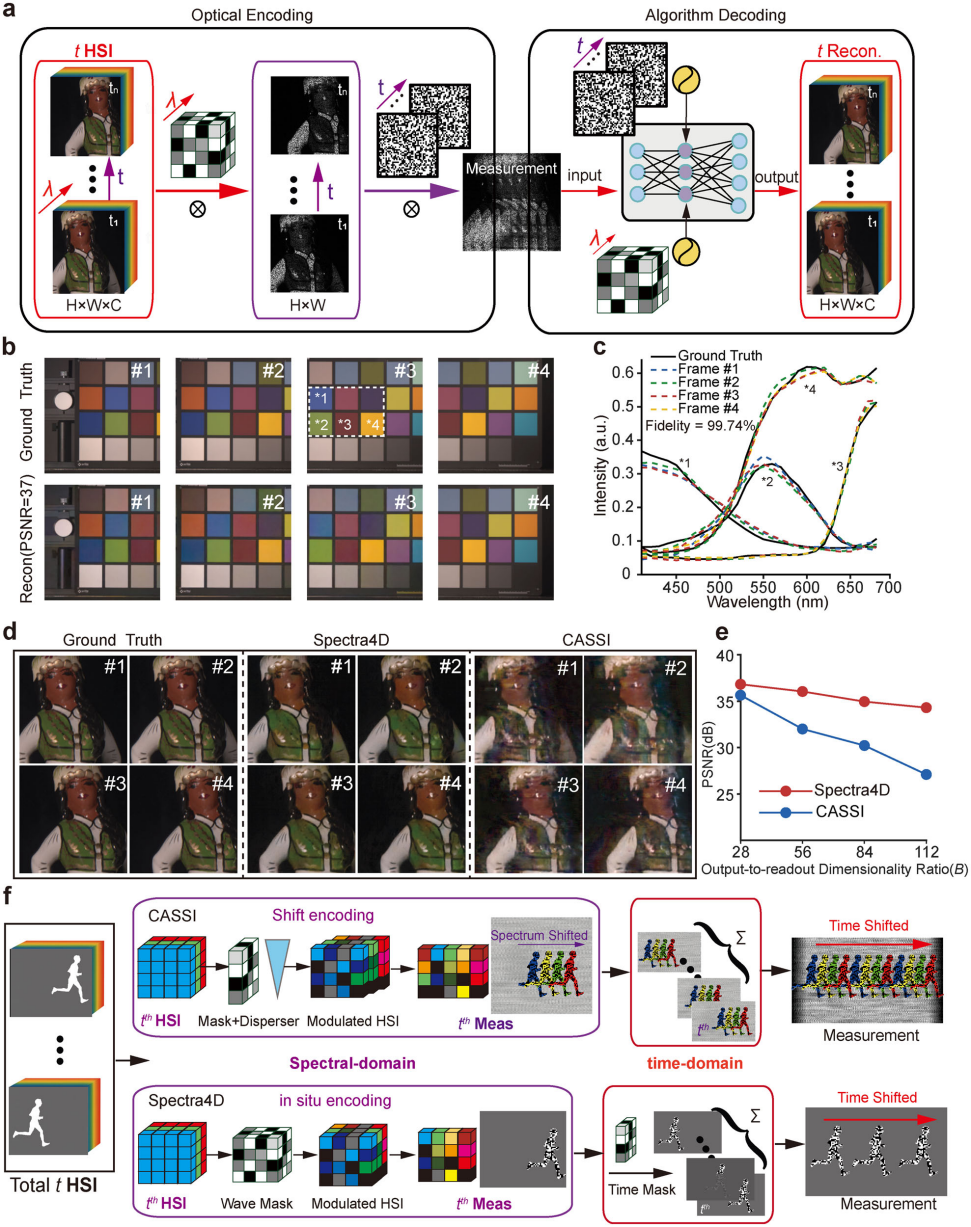
Spectral‐temporal encoding model and reconstructed results of Spectra4D. a Forward model of joint temporal‐spectral multiplexing: the dynamic hyperspectral cube is temporally modulated by DMD masks and spectrally encoded by the on‐chip Fabry‐Pérot microcavity response maps, forming a single 2D snapshot; a schematic decoding flow illustrates recovery of hyperspectral cube using the calibrated dual masks. b Representative simulation results of DM‐NET under joint compression, showing the reconstructed multi‐frame hyperspectral sequence closely matching the ground truth. c Spectral accuracy assessment: spectra extracted from representative spatial regions across multiple recovered frames, comparing ground truth and reconstruction to quantify spectral fidelity. d System‐level comparison with a time‐encoded dispersive CASSI baseline on dynamic scenes, where CASSI exhibits spectral‐temporal aliasinginduced artifacts (edge blurring/color distortions) while Spectra4D better preserves spatial details and spectral consistency. e Quantitative performance versus compression: PSNR comparison between Spectra4D and time‐encoded CASSI with spectral ratio fixed (*C* = 28) and temporal compression varied (*T* = 1–4; total output‐to‐readout dimensionality ratio up to *B* = 112), demonstrating consistently higher reconstruction quality for Spectra4D, especially under extreme joint compression. f Schematic comparison of temporal‐spectral multiplexing in time‐encoded CASSI and Spectra4D. CASSI relies on dispersive spectral shifting, causing wavelength‐dependent spatial displacement to couple with temporal accumulation and leading to spectral‐temporal aliasing. In contrast, Spectra4D uses in situ sensor‐plane spectral encoding, enabling temporal modulation and spectral encoding to remain decoupled for more stable reconstruction.

Reconstruction performance is first evaluated on synthetic dynamic hyperspectral datasets derived from public benchmarks (Figure S5 and Methods). Representative results (Figure 2b and Figure S6) demonstrate that the proposed model can maintain spatial detail and temporal coherence while preserving color consistency across frames. Spectral accuracy is quantified by comparing reconstructed and ground‐truth spectra over four representative spatial regions across multiple time frames (Figure 2c). The recovered spectra closely align with the ground truth in both profile and amplitude, yielding an average spectral fidelity of 99.74%. Here, spectral fidelity is defined as follows:

(4) $$\mathrm{Fidelity}\;=\frac{\sum_{i=1}^{n}\hat{x_{i}}x_{i}}{\sqrt{\sum_{i=1}^{n}{\hat{x_{i}}}^{2}}\sqrt{\sum_{i=1}^{n}x_{i}^{2}}}$$

where n is the number of spectral channels, x_i_ denotes the reference spectrum, and $\hat{x_{i}}$ denotes the reconstructed spectrum. The mean PSNR across the test set reaches 35 dB (Figure S7 and Table S1), and the proposed model outperforms representative hyperspectral reconstruction methods under the same settings (Table S2).

To evaluate the advantages of the proposed Spectra4D system in joint temporal‐spectral reconstruction, we conducted a system‐level simulation comparison against the classical CASSI (Coded Aperture Snapshot Spectral Imaging) architecture. For comparison in a unified framework, we extended the CASSI framework [38, 39, 40, 41, 42] by incorporating temporal encoding into its coded aperture [21, 22, 43, 44, 45, 46, 47, 48] (Methods). Representative reconstructions from four frames are shown in Figure 2d. It can be observed that CASSI suffers from noticeable spatial and spectral distortions, whereas Spectra4D better preserves structural details and spectral consistency, and the reconstructions are closer to the ground truth. Quantitative results under varying nominal output‐to‐readout dimensionality ratios are summarized in Figure 2e and Figure S8. With spectral ratio fixed at 28 and temporal ratio varied from 1 to 4, Spectra4D consistently achieves higher PSNR across all settings. Notably, under extreme compression, Spectra4D still retains a clear quantitative advantage (Figures S8 and S9 and Tables S3 and S4).

The essential difference lies in the spectral encoding mechanism before temporal accumulation (Figure 2f). In CASSI, spectral encoding is achieved through dispersion, which causes different wavelengths to be shifted to different sensor positions. When measurements from multiple temporal states are further integrated within a single exposure, wavelength‐dependent spatial displacement and temporal superposition become coupled on the detector, resulting in pronounced spectral‐temporal aliasing under high compression. By contrast, Spectra4D performs spectral encoding directly on the sensor plane using a spatially registered micro‐filter array, so different wavelengths are sampled at fixed pixel locations without dispersion‐induced spatial shift. Temporal modulation is then applied independently, and the final compressed measurement is formed without additional spectral displacement. This decoupled encoding strategy reduces overlap in the multiplexed measurement and improves reconstruction stability, consistent with the reduced artifacts and higher fidelity observed in Figure 2d,e.

### 2.3 Quantitative Evaluation of Spectra4D in the Physical System

Next, we characterize the experimental performance of Spectra4D by evaluating its spectral fidelity and spatial resolution (Methods and Figure S10). Spectral calibration was performed using a standard 24‐color ColorChecker. As dynamic ground truth is not directly accessible, a static hyperspectral reconstruction without temporal coding is used as reference. During dynamic acquisition, only the target position is varied, while its orientation, reflectance, and imaging geometry remain unchanged under highly stable illumination. The temporally reconstructed frames remain consistent with the reference in both spatial and spectral characteristics (Figure 3a). Quantitative spectral accuracy is further evaluated using spectrometer (USB2000+, Ocean Optics) measurements by comparing reconstructed spectra from the central regions of 12 representative color patches (Figure 3a) with spectrometer‐measured ground truth. The average spectral fidelity reaches 96.47% for the four temporally compressed reconstructions (Figure 3b and Figure S11). Analysis of patches 1–4 shows that the spectral similarity across time frames exceeds 99%, and the intensity error is less than 5.5% in the low‐signal region where the relative intensity is less than 0.2. The lower fidelity in the blue patches (e.g., patches 1 and 8, λ < 480 nm) arises from the limited blue‐band output of the halogen illumination source (Figure S10), leading to reduced signal‐to‐noise ratio and degraded spectral accuracy. An additional spectral‐resolution characterization using narrowband illumination demonstrates resolving capability sufficient to support the 10 nm interval of the 28‐channel configuration (Figure S12). These results demonstrate that Spectra4D achieves a fourfold increase in temporal resolution with minimal loss in spectral fidelity.

**FIGURE 3:**
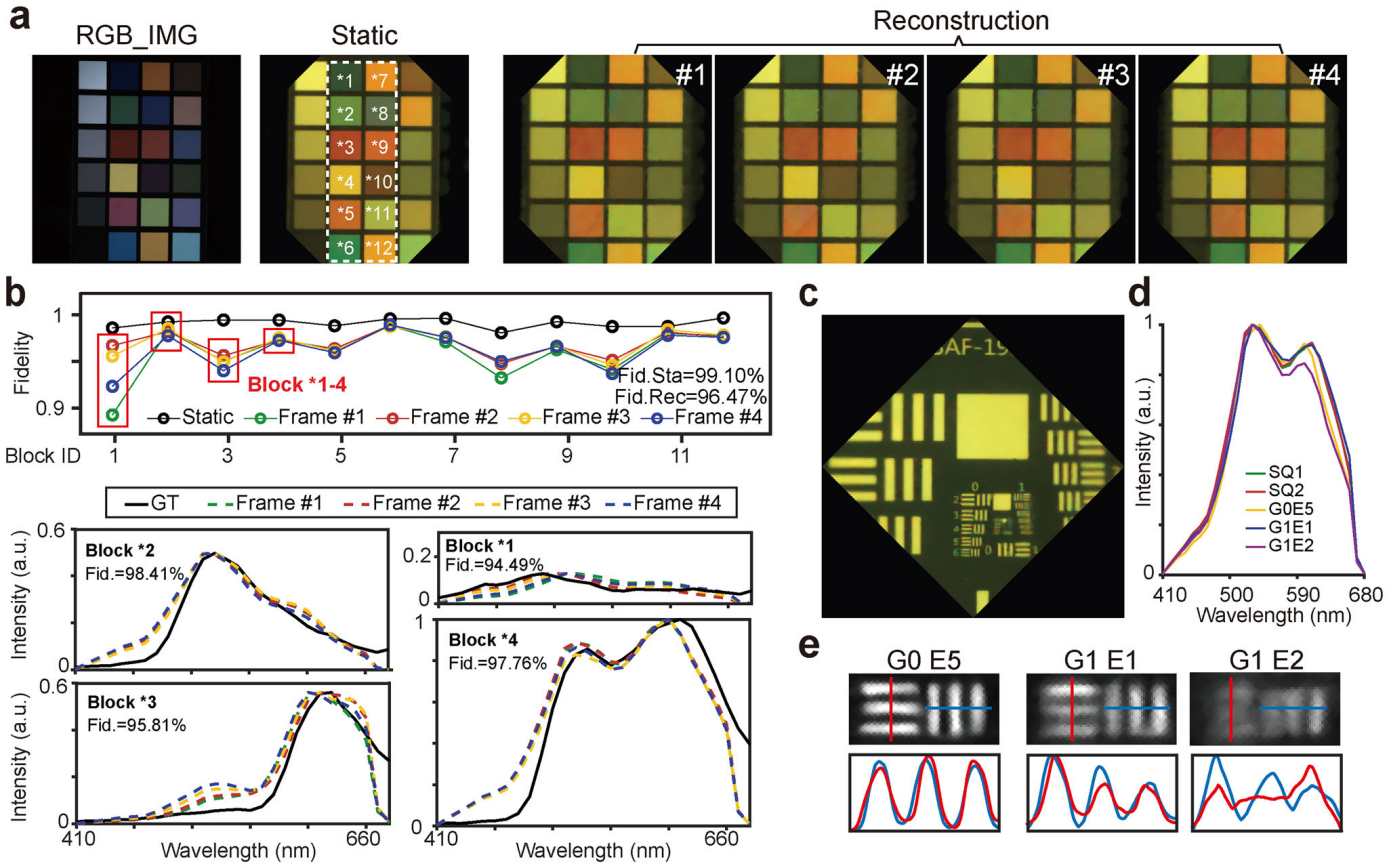
Experimental characterization and performance validation of the Spectra4D. a Dynamic ColorChecker experiment for spectral‐temporal characterization. A static 28‐band hyperspectral reconstruction without temporal coding is used as the spectral reference, while under stage motion and temporal coding a single compressed snapshot is decoded into four hyperspectral frames, visualized here as synthesized RGB images. b Spectral fidelity evaluation on 12 representative ColorChecker patches. Top: patch‐wise fidelity statistics for the static reference and the four reconstructed frames. Bottom: representative reconstructed spectra for blocks 1–4, showing close agreement across temporally recovered frames. c Reconstructed pseudocolor image of a USAF 1951 resolution target for spatial‐resolution characterization. d Reconstructed spectra extracted from representative regions of the USAF target, showing high spectral consistency across different spatial locations. e Enlarged views and corresponding line profiles for selected USAF elements (G0 E5, G1 E1, and G1 E2), demonstrating preserved contrast for fine spatial features and a resolvable spatial frequency up to approximately 2 lp mm^−1^.

The spatial resolution of the Spectra4D system was evaluated using a USAF (United States Air Force) 1951 target, under the same illumination conditions and compression ratio as the ColorChecker experiment. The reconstructed pseudocolor image (Figure 3c and Figure S13) preserves both the overall geometry of the target and the local high‐frequency line structures, and spectra extracted from different regions also show stable profiles and peak positions (Figure 3d). For the spatial resolution, Spectra4D can clearly resolve Group 1 Element 1, corresponding to a spatial frequency of 2 lp mm^−1^ (Figure 3e), demonstrating reliable spectral reconstruction for objects with fine spatial structures.

### 2.4 Snapshot Reconstruction of Dynamic Spatio‐Spectral Videos

We next evaluate the performance of the Spectra4D system on time‐varying spatio‐spectral video content that is not designed for calibration or benchmarking. These targets exhibit varying spectral composition, fine spatial structure, and temporal evolution, thereby providing a more representative assessment of practical snapshot hyperspectral video reconstruction. As shown in Figure 4a, a planar target with smooth spectral transitions and sharp text features is used to examine the preservation of spectral continuity and spatial edges under temporal compression. In contrast, Figure 4c presents 3D objects with nonuniform surfaces and textured features undergoing dynamic motion.

**FIGURE 4:**
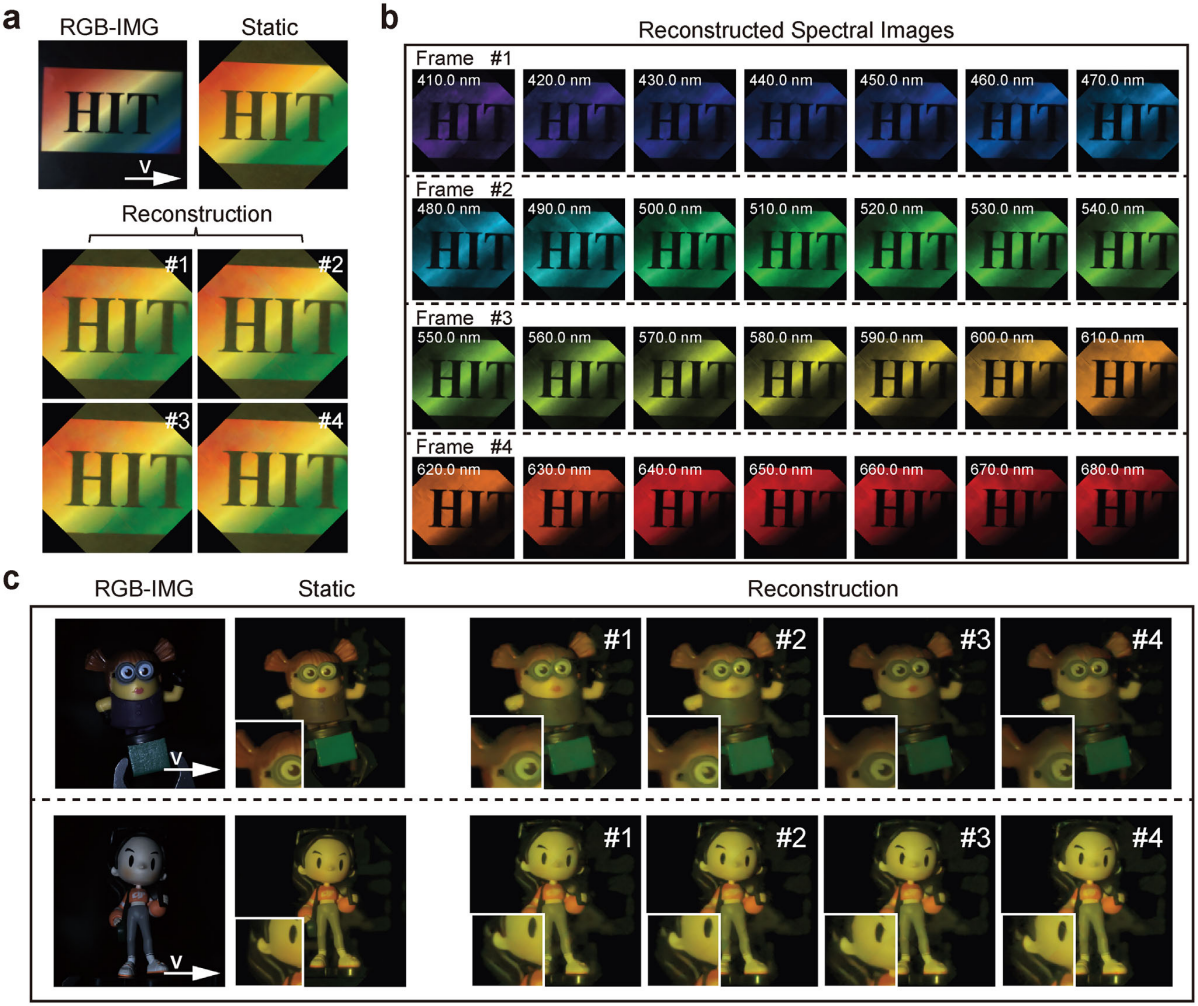
Spatio‐spectral video reconstruction in dynamic scenes with diverse spatial and spectral structures. a, b Reconstruction of a planar target with continuous color gradients and the embedded text ‘HIT’, illustrating spectral fidelity, edge preservation, and temporal consistency across multiple spectral bands and temporal frames. c Hyperspectral reconstruction results of two animated character models with complex 3D geometries, demonstrating stable temporal tracking and accurate spectral reconstruction under high‐speed motion conditions.

The design of the experimental platform is described in the Methods section. As in the previous experiments, a static snapshot without temporal compression was first reconstructed as the spectral reference, followed by time‐resolved hyperspectral reconstruction under a four‐fold temporal compression. For the planar target (Figure 4a), the reconstructed hyperspectral images faithfully recovered the continuous spectral transition of the background from red to green, together with the sharp boundaries of the text features (Figure 4b and Figure S14). For the dynamically moving 3D target (Figure 4c and Figure S15), the reconstructed frames accurately captured inter‐material spectral differences while retaining fine structural details and spatially heterogeneous spectral responses, including the local structures of the doll's eyes and the texture variations on the hair surface.

### 2.5 High‐Frame‐Rate Hyperspectral Imaging of Dynamic Fluorescent Microspheres

Dynamic fluorescence microsphere imaging represents a generic imaging for a broad class of fluorescence‐based measurements of moving point emitters. Such measurements arise commonly across applications including protein tracking, fluid flows analyzing, and drug screening [49, 50, 51]. Constrained by temporal resolution, existing multispectral imaging methods are struggled with motion blur and unreliable trajectory connection. To demonstrate the capability of Spectra4D, we applied it to monitor the motions of two fluorescent microspheres with distinct radii (10 and 15 µm) and emission spectra (Cy3 labeled, 606 nm; FITC labeled, 526 nm) in aqueous solution.

We built a controlled microscopic imaging platform (Figure S16) to track the dynamic behavior of two spectrally distinct microspheres. The microspheres were immobilized on a glass slide and translated using a motorized stage to generate repeatable and calibrated motion. Notably, no fluorophore‐specific spectral filters or separate detection channels were employed in this system, and spectral discrimination between the two microsphere species was achieved solely from the reconstructed spectra of Spectra4D. During the experiment, images were acquired at 50 fps over 10 consecutive frames. Owing to the rapid motion of the microspheres, conventional imaging without temporal encoding leads to pronounced motion blur and localization inaccuracy (Figure 5c). In contrast, under 4:1 temporal compression, Spectra4D decodes each single exposure into a temporally coherent four‐frame sequence, effectively reducing motion‐induced artifacts and improving localization fidelity (Figure 5a–c and Movie S1). The reconstructed spectra of both fluorophores closely match their reference spectra in terms of spectral shape and peak position across all recovered frames (Figure 5d and Figure S17). Joint kernel density estimation of particle radius and peak wavelength reveals two clearly separated clusters (Figure 5e and Note S2). The first population exhibits a mean peak wavelength of 525.1 ± 1.8 nm and an average particle radius of 15.7 ± 1.2 µm, while the second population centers at 605.9 ± 5.9 nm with a radius of 9.8 ± 0.5 µm. The inter‐cluster separation along the spectral axis is approximately 81.7 nm, confirming reliable spectral discrimination between the two microsphere species.

**FIGURE 5:**
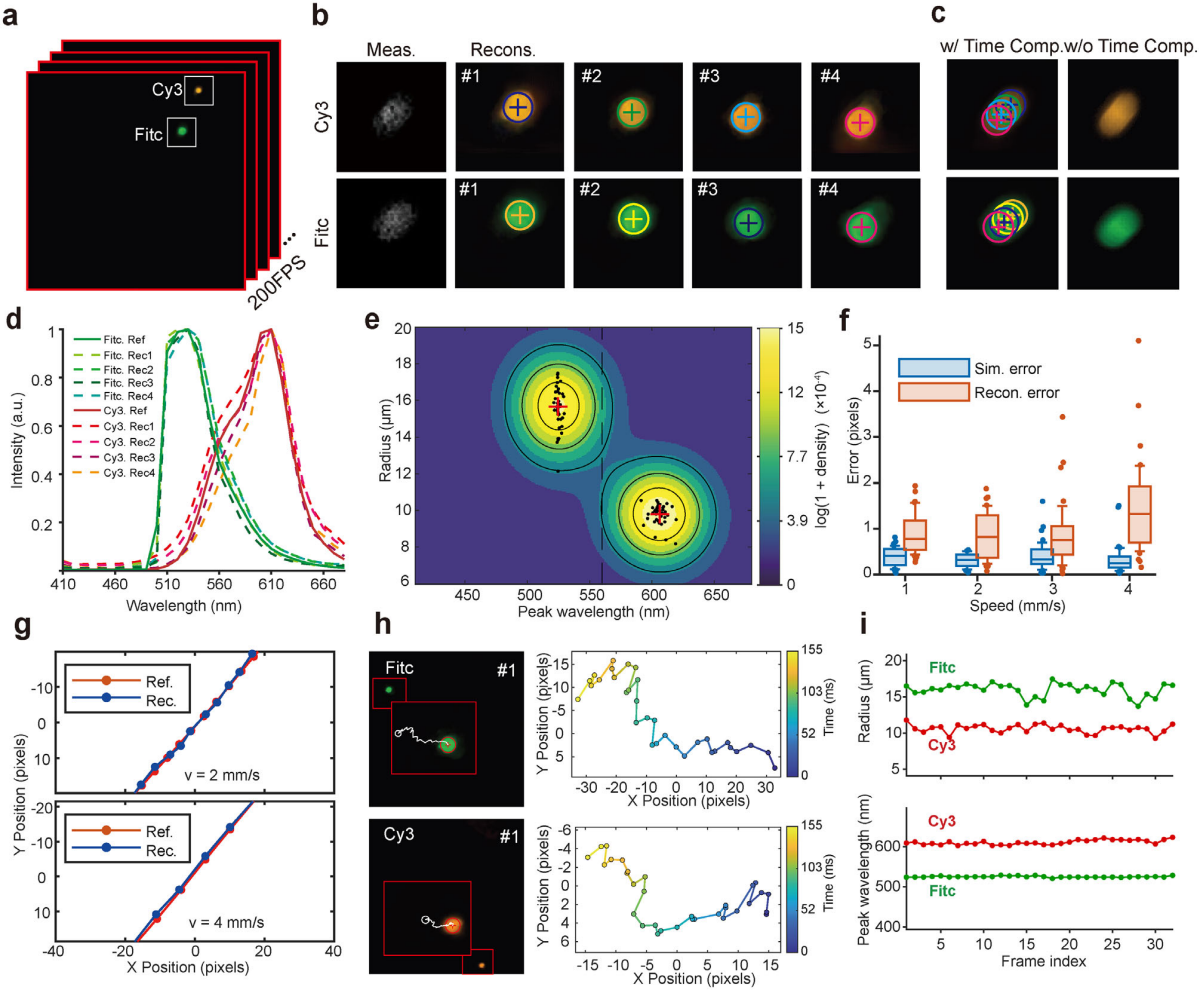
High‐frame‐rate hyperspectral imaging and tracking of fluorescent microspheres. a Wide‐field reconstruction of two fluorescent microsphere populations at an effective temporal sampling rate of 200 fps, showing consistent separation of the two types across frames. b Single‐shot measurement and decoded four‐frame hyperspectral reconstructions for Cy3 and FITC, demonstrating snapshot recovery of a time‐resolved spectral sequence. c Comparison of reconstructions with and without temporal compression, where temporal encoding suppresses motion‐induced blur and localization bias. d Spectral identity preservation during tracking: reference spectra and reconstructed spectra of FITC and Cy3 across the recovered four frames, showing consistent spectral shape and peak position. e Joint kernel density estimation (KDE) of peak emission wavelength versus particle radius aggregated over tracking frames, yielding two well‐separated clusters for robust fluorophore discrimination. f Spatiotemporal localization accuracy quantified at four target speeds in simulation and experiment (localization error of reconstructed bead centers). g Overlay of reference and reconstructed bead centers for two representative speeds, illustrating accurate trajectory recovery even at the highest tested speed. h Dynamic hyperspectral reconstruction of microspheres suspended in a flow field (two samples), showing reconstructed trajectories under complex motion backgrounds. i Frame‐wise evolution of extracted peak wavelength and radius from the flow‐field reconstructions, confirming stable spectral identity and consistent size estimation over time.

To quantitatively evaluate the influence of motion blur on reconstruction accuracy, we conducted experiments at four translational speeds while maintaining a constant camera frame rate of 50 fps. For each speed, microsphere centers were localized from the experimentally reconstructed frames and compared against the corresponding ground‐truth positions provided by the calibrated motorized stage, while simulated reconstructions were evaluated in parallel for supplementary comparison (Figure 5f and Methods). The mean localization error remained at 0.87 ± 0.20 pixels for low speeds and increased to 1.423 ± 0.96 pixels at the highest tested speed. Even at the fastest motion, the reconstructed trajectories closely matched the reference paths (Figure 5g). These results indicate that Spectra4D maintains stable spatiotemporal localization accuracy under increasing motion, with the mean error remaining below 1.5 pixels across the entire tested speed range.

Finally, we evaluated the reconstruction stability of Spectra4D under complex 2D motion by imaging microspheres freely suspended in solution (Figure 5h and Movies S2 and S3). Unlike the calibrated translational motion in previous experiments, the suspended beads exhibited stochastic planar trajectories with continuously varying velocity and direction. Frame‐wise analysis shows that the peak emission wavelength remains at 610.9 ± 5.8 nm / 525.2 ± 1.7 nm and the estimated particle radius fluctuates within 5.4% / 5.8% across the sequence, indicating stable spectral identity and radius estimation despite the dynamic background (Figure 5i). These results demonstrate that Spectra4D maintains robust spatiotemporal‐spectral reconstruction accuracy even under complex 2D motion.

### 2.6 High‐Frame‐Rate Elemental Monitoring in Combustion

Owing to element‐specific electronic transitions, different elements exhibit distinct spectral responses during combustion. For example, sodium combustion produces a characteristic yellow emission, while lithium exhibits prominent red emission features. To visualize these element‐dependent spectral responses in dynamic flames, Spectra4D was next applied to frame‐resolved quantitative monitoring of flame combustion. Specifically, we captured hyperspectral images of ethanol flames at a temporal compression ratio of 4:1 under three conditions:undoped, Na‐doped, and Li‐doped (Figure 6a). Representative images at three characteristic wavelengths (550, 590, and 670 nm) and corresponding pseudo‐color reconstructions under different doped conditions reveal distinct spatial distributions of elemental emission within the flame. The reconstructed spectra closely match measurements from a commercial spectrometer (Figure 6b), with the spectral fidelity exceeding 99.1%. These results confirm that Spectra4D accurately resolves elemental spectral features that cannot be reliably distinguished by RGB imaging alone (Figures S18–S20).

**FIGURE 6:**
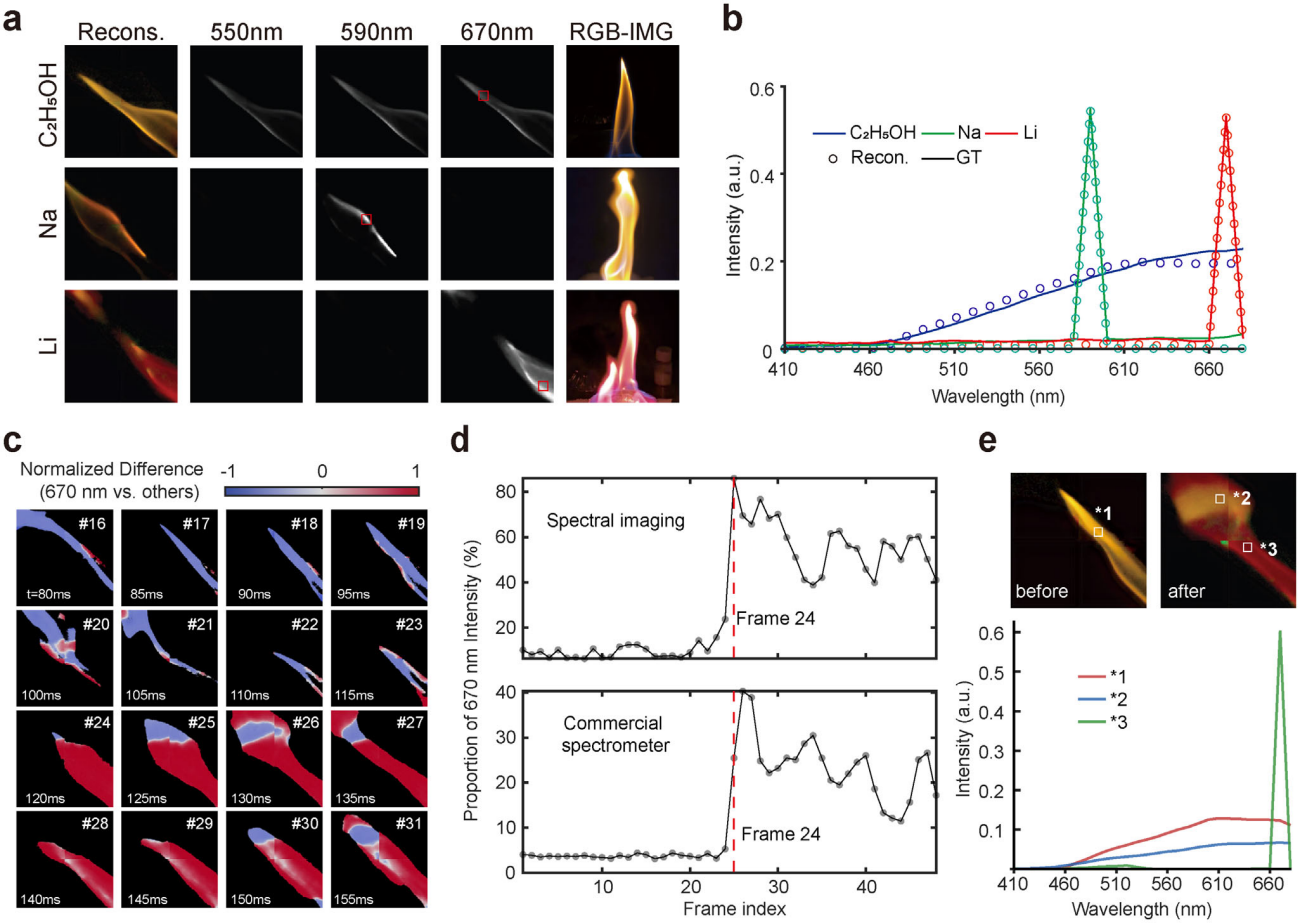
High‐frame‐rate elemental diagnostics in dynamic ethanol flames enabled by Spectra4D. a Representative reconstructions of undoped, Na‐doped, and Li‐doped ethanol flames acquired under 4:1 temporal compression (four recovered frames per snapshot), shown as pseudocolor hyperspectral renderings together with narrowband images at 550, 590, and 670 nm and the synthesized RGB for direct spatial comparison of band‐dependent radiance redistribution. b Spectra extracted from the reconstructed datacubes under the three conditions, compared against a synchronously triggered commercial spectrometer, highlighting distinct elemental emission signatures. c Frame‐resolved band‐normalized difference maps at 670 nm during Li injection, visualizing the onset and spatial expansion of the Li‐related spectral perturbation across consecutive recovered frames. d Temporal evolution of the 670 nm energy proportion estimated from Spectra4D reconstructions and the reference spectrometer, showing a consistent transition at the Li‐injection moment (e.g., around frame 24). e Spatially resolved reconstructions before and after injection with selected ROIs and their spectra, revealing the coexistence of Li‐dominated and ethanol‐dominated flame components and a transitional zone that cannot be captured by spatially integrated spectroscopy alone.

Next, we introduced lithium into an ethanol flame to test whether Spectra4D can capture elemental dynamics at high frame rates. For quantitative comparison, a synchronously triggered spectrometer was operated in parallel as an independent reference. The flame initially exhibits spectral signatures dominated by ethanol combustion. At a specific time point, lithium is injected into the flame, introducing a rapid and localized spectral disturbance. The intensity ratio between 670 nm and the remaining spectral channels indicates that the emergence of lithium emission is clearly resolved as a sharp, frame‐resolved transition in both spectral intensity and spatial distribution, thereby enabling direct identification of elemental emission components in the ethanol flame (Figure 6c and Figure S21). In particular, a rapid enhancement of the lithium characteristic line is observed immediately after injection (Movie S4), in quantitative agreement with measurements from a commercial spectrometer (Figure 6d). Importantly, the spatially resolved reconstructions reveal the distribution of lithium‐dominated regions within the flame (Figure 6e). These regions coexist with ethanol‐dominated components and are separated by a transitional interface, highlighting a heterogeneous combustion state that cannot be resolved by spatially integrated spectroscopy alone.

## 3 Discussion

In summary, we developed Spectra4D, a dual‐domain compressive snapshot spatiotemporal–spectral sensing platform that reconstructs HSI with up to 28 spectral bands at an effective temporal sampling rate of 200 fps on a near‐megapixel spatial grid (with spatial resolution comparable to bare‐CMOS imaging, Figure S22). By decoupling on‐chip spectral encoding from DMD‐based temporal modulation, Spectra4D suppresses spectral‐temporal interference and enables high‐fidelity recovery of both spectral and temporal information. This proposed strategy substantially increases the effective measurement bandwidth and alleviates the long‐standing trade‐off among temporal, spatial, and spectral resolution in hyperspectral imaging.

By unifying spatial, temporal, and spectral sensing within a single snapshot framework, Spectra4D paves the way for hyperspectral imaging of dynamic systems. Beyond combustion demonstrations, this unified sensing capability opens opportunities for studying fast, multi‐parameter dynamics across biological, chemical, and material systems. With continued advances in on‐chip optical encoding and physics‐constrained reconstruction, multi‐domain encoded snapshot imaging could be extended to the joint measurement of additional physical dimensions, including phase, polarization, and depth, thereby establishing a general platform for high‐dimensional quantitative imaging of complex dynamic processes.

## 4 Methods

### 4.1 Fabrication of the On‐Chip Encoded Spectral Camera

#### 4.1.1 Fabrication of the Micro‐Filter Array

The fabrication of the micro‐filter array is executed on a 1‐mm‐thick fused silica substrate. Initially, a five‐layer bottom reflector and a sacrificial layer are deposited using an ion‐assisted electron beam evaporation system (GTV‐1150, CHENGDU GUOTAI). To achieve the spatially varying topography, a single‐step grayscale lithography process (PicoMaster ATE‐100, Raith) is performed on a spin‐coated AZ 1500 photoresist layer to define a periodic photoresist array. This relief pattern is subsequently transferred to the sacrificial layer via inductively coupled plasma (ICP) etching, yielding a periodic intermediate cavity layer. The fabrication process concludes with the deposition of a final five‐layer top reflector, resulting in an 11‐layer architecture. The completed array comprises 16 F‐P cells arranged in a 4×4 periodic and tiled across the active area. Each F‐P cell measures 7.2×7.2 µm^2^, corresponding to approximately 3×3 CMOS pixels.

#### 4.1.2 Integration of the Nanophotonic Film Array on the CIS (CMOS Image Sensor)

The cover glass of the monochromatic CMOS image sensor (Hikrobot, MV‐CB016‐10UM‐C with Sony IMX273 sensor and MV‐CB060‐10UM‐C with Sony IMX178 sensor) is initially removed. The micro‐filter arrays are mechanically cut into 5 mm×5.5 mm pieces, and edge bonded to the sensor using the optical adhesive with the aid of the transfer platform. In this assembly, the top reflector and the sensor surface are oriented face‐to‐face to minimize the physical gap between the spectral encoding layer and the detection plane. This proximity coupling effectively suppresses lateral light scattering and reduces inter‐pixel crosstalk.

### 4.2 Training and Testing Datasets

Since there are no publicly available datasets specifically designed for dynamic multi‐frame hyperspectral imaging, we constructed synthetic dynamic hyperspectral training and testing datasets based on existing static datasets. Specifically, 30 scenes from the CAVE [52] dataset and 11 scenes from the KAIST [53] dataset were used to construct the synthetic training and testing datasets, respectively. To simulate temporal dynamics, we extracted multiple sub cubes of size 384×384×28 by performing evenly spaced horizontal cropping along the central rows of each image. These sub‐cubes were then arranged in left‐to‐right order to form a sequence, which was treated as a multi‐frame hyperspectral dataset corresponding to a dynamic scene. Additional data augmentation was employed for the flame‐emission and fluorescent‐microsphere experiments (Note S1).

### 4.3 Comparison with Temporally Extended CASSI

To benchmark Spectra4D under joint spectral‐temporal compression, we constructed a temporally extended CASSI model within the same simulation framework (Figure 2f). The same dynamic hyperspectral scenes were used as inputs for both systems, while the spectral channel number, temporal compression ratio, and total nominal output‐to‐readout dimensionality ratio were matched. Specifically, both models used 28 spectral channels and four temporally coded frames. In the temporally extended CASSI baseline, each temporally coded hyperspectral frame was encoded by the mask and disperser, yielding a corresponding spectrum‐shifted measurement for that temporal state. The final compressed measurement was obtained by integrating these temporally distinct measurements on the detector. In Spectra4D, the same inputs were spectrally encoded by the calibrated wave mask of the on‐chip micro‐filter array and subsequently modulated by the time mask before detector integration (Figure 2f).

### 4.4 Experimental Setup

#### 4.4.1 Quantitative Evaluation of Spectra4D System

The Spectra4D system is calibrated and tested in experiments. The target is mounted on a motorized linear translation stage and actuated by an electric motor to simulate dynamic scenes. The moving target is imaged through a commercial imaging lens and a telecentric lens onto the DMD (FLDiscovery F4320 DDR 0.95 1080P), then reflected and relayed by a second telecentric lens onto the monochrome CIS (Figure S10). A micro‐filter array fabricated with an asymmetric weak F‐P cavity structure is directly integrated on the surface of the CIS to perform spectral modulation. Illumination is provided by a halogen lamp source, spectrally constrained by a 400–700 nm bandpass filter (Figure S10). The temporal masks and F–P spectral responses are calibrated pixelwise on the CIS grid (Figure S23). By employing the proposed temporal‐spectral reconstruction algorithm, the Spectra4D system can reconstruct four frames of 864×864×28 hyperspectral images from a single compressed measurement.

To obtain a high‐quality reference for subsequent evaluation, we first captured the static scene without applying temporal compression, with the DMD held fully open during acquisition. A single hyperspectral frame comprising 28 spectral bands was reconstructed as reference, with the channels uniformly distributed across the 410–680 nm spectral range. Based on the colorimetric theory of the Commission Internationale de l’Éclairage (CIE) [54], the hyperspectral image was converted into a synthetic RGB image for visualization. Next, we activated the motorized platform and applied binary masks to the DMD for temporal modulation. Corresponding synthetic RGB images were converted from the reconstructed hyperspectral data.

#### 4.4.2 Elemental Monitoring in Flame Combustion

The optical system followed the same configuration as the quantitative evaluation platform, except that the external illumination source and the translation‐stage motion actuation were removed, and the flame self‐emission was used as the illumination source. For quantitative comparison, an independent reference spectrometer was operated in parallel with an integration time of 2.5 ms, while both the spectrometer and CMOS camera were hardware‐triggered by the DMD to ensure precise temporal synchronization.

#### 4.4.3 Fluorescent Microsphere Hyperspectral Imaging

Cy3 fluorescent microspheres (10 µm radius) and FITC fluorescent microspheres (15 µm radius) were immobilized on a glass slide, with repeatable, calibrated translational motion introduced by a motorized stage (Figure S16). Localization accuracy was quantified using both experimental and simulated benchmarks. In the experimental benchmark, 10 temporally compressed measurements were acquired under the prescribed motion condition, yielding 40 bead‐center estimates after reconstruction. To generate ground‐truth positions along the same trajectory, the stage repeated the motion path at one‐quarter of the original speed, with temporal compression disabled while keeping the camera frame rate and all other imaging conditions unchanged. Under this setting, 40 directly captured frames were obtained and used as the ground truth for frame‐by‐frame localization‐error analysis. In parallel, a simulation benchmark was constructed by digitally synthesizing compressed measurements from sequential frames using the temporal masks, thereby establishing a controlled forward model with known ground truth. This simulation provides a reference for the achievable localization accuracy under idealized encoding and reconstruction conditions. The IMX273 sensor, with its larger pixel size, was used for the flame‐emission and fluorescent‐microsphere experiments to improve photon‐collection efficiency, whereas the IMX178 sensor was used for other experiments.

## Author Contributions

C.C. initiated the project. C.C., J.W., Y.Z., and H.S. conceived of the project. C.C., J.W., and H.S. conducted the experiments and analyzed the data. J.W. and C.Y. contributed to the fabrication of the spectral camera and data analysis. C.C. contributed to sample preparation. C.C., H.S., and Y.Z. wrote the manuscript. G.L. and Y.Z. supervised the project.

## Conflicts of Interest

The authors declare no conflicts of interest.

## Code Availability

The code that supports the findings of this study is available from the corresponding author upon reasonable request.

## Supporting information




**Supporting File 1**: advs78383‐sup‐0001‐SuppMat.pdf.

**Supporting File 2**: advs78383‐sup‐0002‐MovieS1.mp4.

**Supporting File 3**: advs78383‐sup‐0003‐MovieS2.mp4.

**Supporting File 4**: advs78383‐sup‐0004‐MovieS3.mp4.

**Supporting File 5**: advs78383‐sup‐0005‐MovieS4.mp4.

## Data Availability

The data that support the findings of this study are available from the corresponding author upon reasonable request.
