# Supplementary material for: Snapshot Spatiotemporal‐Spectral Imaging Enabled by Joint Photonic Encoding
Source: Adv Sci (Weinh). 2026 Oct 22:e78383. Online ahead of print. doi: 10.1002/advs.78383 (PMC13690980; doi:10.1002/advs.78383)
Supplement: Supplementary file 1 [file ADVS-9999-e78383-s003.pdf]

Supplementary Information

Snapshot Spatiotemporal-Spectral Imaging Enabled by Joint Photonic Encoding

Chen et al.

Supplementary Figures

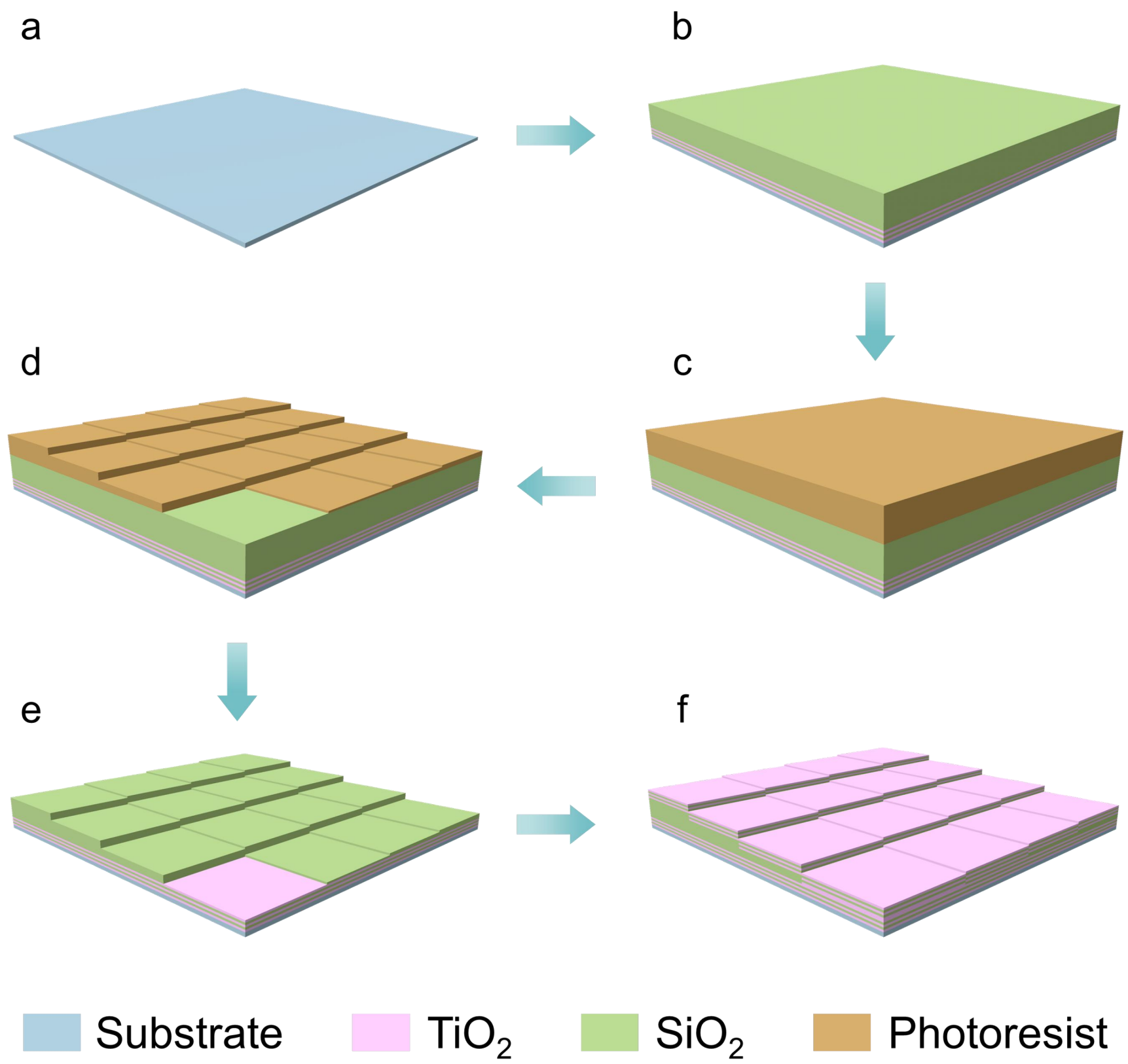

Supplementary Figure 1. Fabrication of the Fabry-Pérot (F-P) micro-filter array. (a) Substate cleaning. **(b)** Deposition of the five-layer bottom reflector and the intermediate layer using electron beam evaporation. **(c)** Spin-coating of the photoresist. **(d)** Patterning of the photoresist using single-step grayscale lithography to form a 4×4 stepped relief pattern. **(e)** Pattern transfer of the stepped profile into the underlying intermediate layer via inductively coupled plasma (ICP) etching. **(f)** Deposition of the five-layer top reflector complete the F-P micro-filter array.

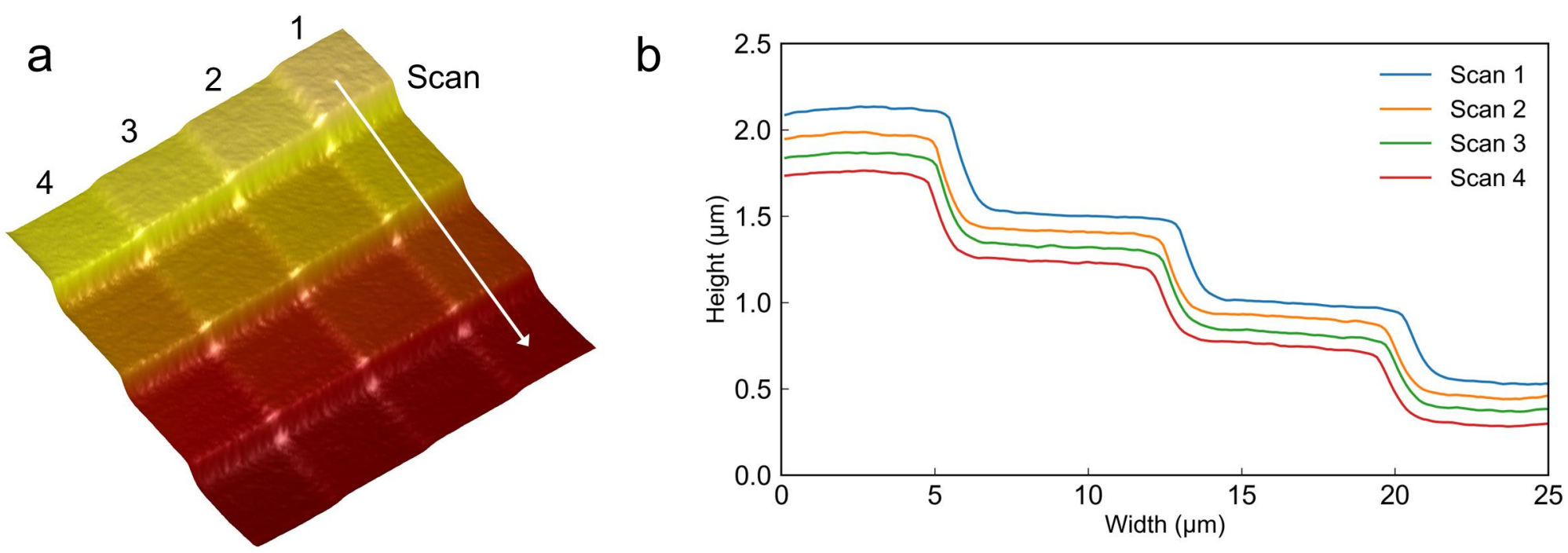

Supplementary Figure 2. Atomic Force Microscopy (AFM) characterization of the F-P micro-filter array. **(a)** Three-dimensional AFM surface morphology image of the fabricated 4×4 micro-array. The white arrow indicates the scanning direction across the array to obtain the cross-sectional profiles in **(b)**. **(b)** Measured cross-sectional height profiles along the four distinct scanning paths (Scan 1-Scan 4) indicated in **(a)**. The curves clearly demonstrate the well-defined stepped structure with varying total heights.

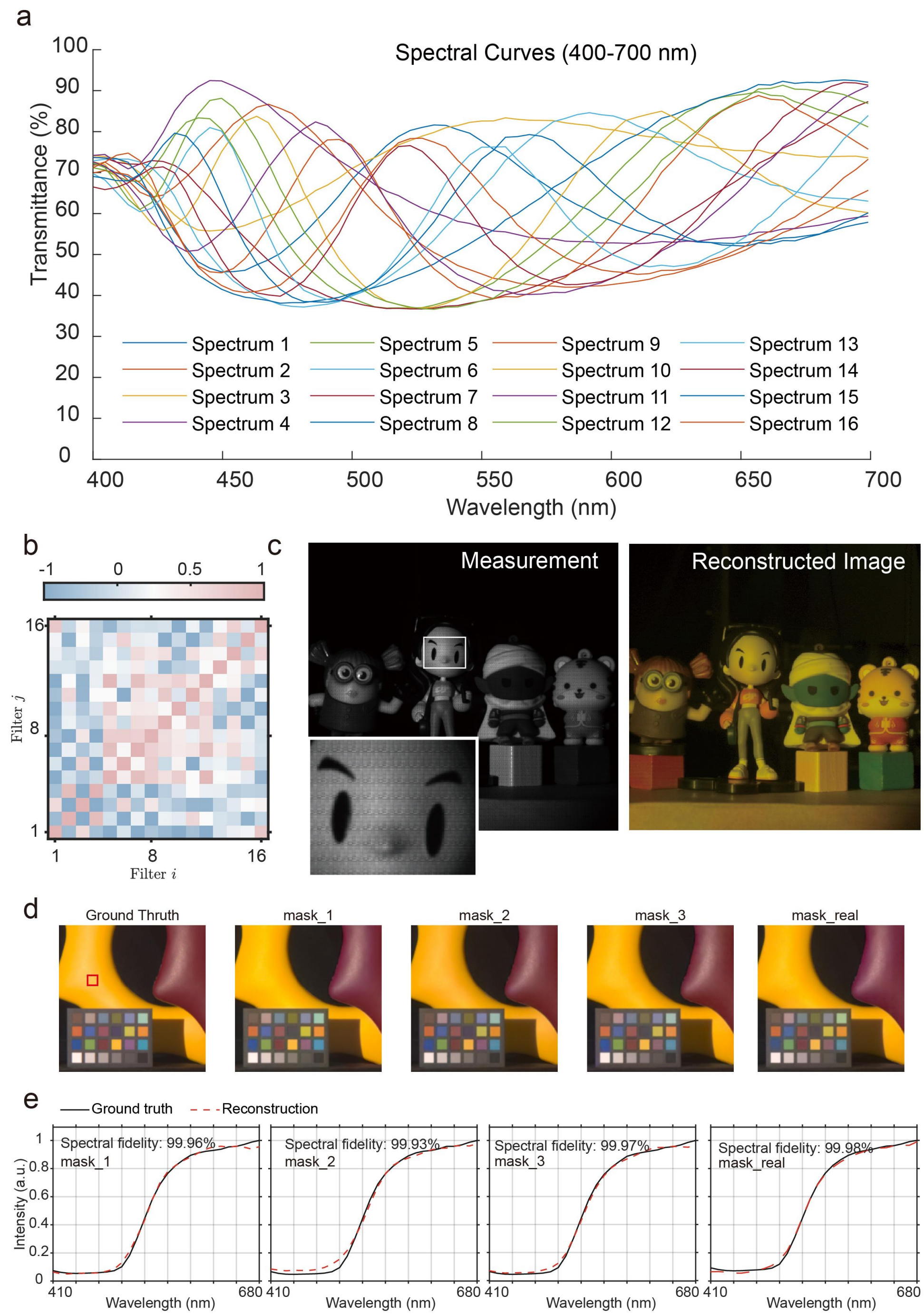

Supplementary Figure 3. **Spectral characterization of the 16-channel filter set and representative reconstruction result. (a)** Measured transmission

spectra of the 16 filters over the 400 – 700 nm range. The filter responses exhibit distinct wavelength-dependent transmission profiles, providing the spectral encoding basis for multi-channel image acquisition. **(b)** Pearson correlation coefficient matrix of the 16 filter spectra, showing the pairwise spectral similarity between different filters. The overall low off-diagonal correlation indicates that the filter set provides sufficiently diverse spectral responses for effective spectral discrimination and reconstruction. **(c)** Representative grayscale measurement acquired by the encoded imaging system and the corresponding reconstructed image. The result demonstrates that the characterized filter set supports practical spectral encoding and subsequent image reconstruction in a real scene. **(d)** Ground truth and reconstruction results obtained using ideal 1×1, 2×2, and 3×3 masks and the experimentally calibrated real mask. The three ideal masks were constructed from the same 16 measured F-P responses, with each response uniformly repeated over a 1×1, 2×2, or 3×3 CMOS-pixel region. The real mask directly used the pixelwise calibrated responses of the fabricated device, including intra-cell nonuniformity and boundary-transition effects. All configurations were evaluated using the same forward model, datasets, training protocol. The corresponding PSNR values were 38.10, 37.97, 37.42, and 37.77 dB, respectively. **(e)** Reference and reconstructed spectra extracted from the marked region in **(d)**. Boundary-transition and alignment-induced nonuniformities give the measured mask pixel-level coding diversity, enabling performance comparable to the ideal 1×1 mask.

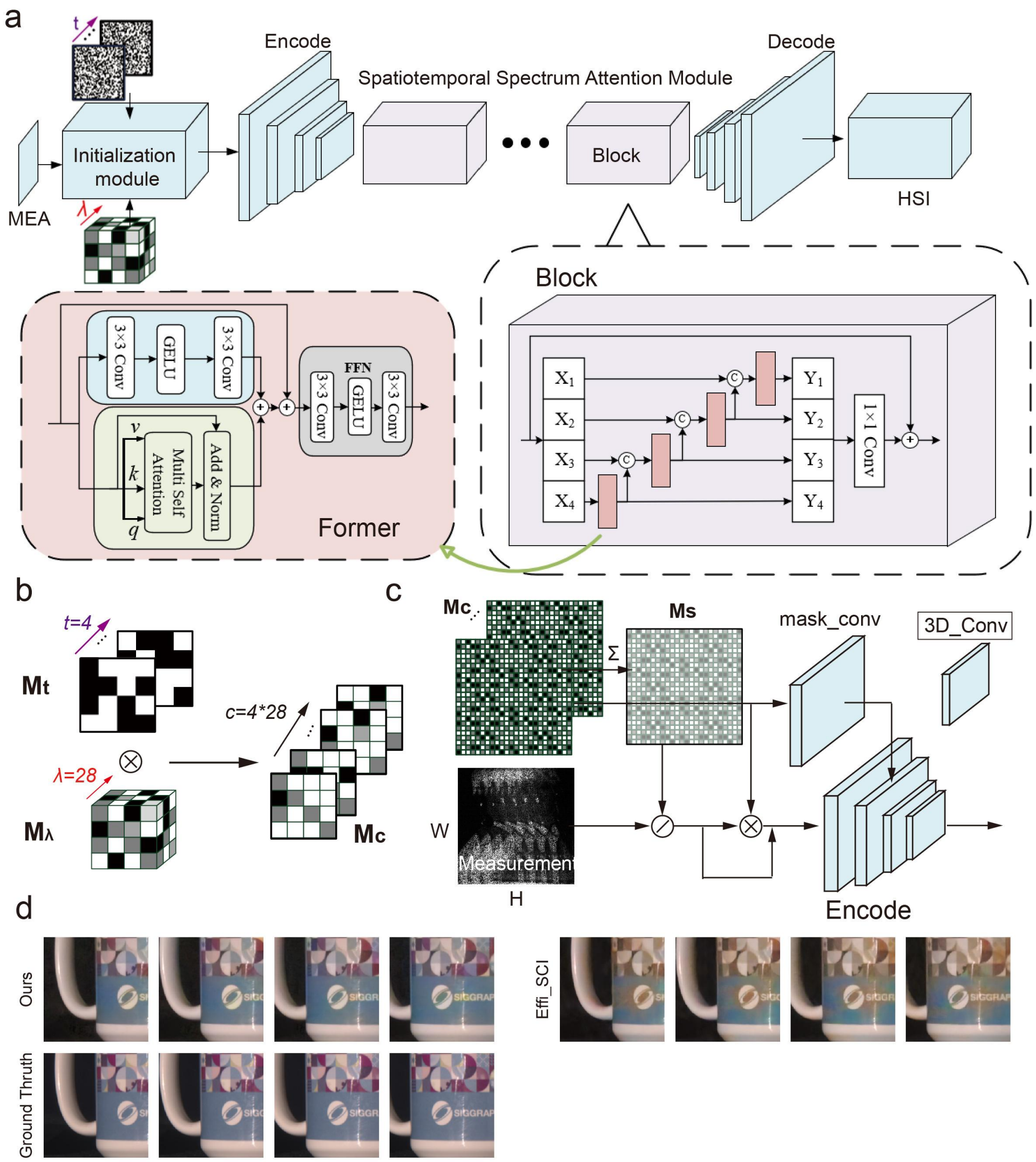

Supplementary Figure 4. Architecture of the proposed DM-Net for dynamic hyperspectral reconstruction. (a) Overall architecture of DM-Net and structures of the basic Block and Former modules. The compressed measurement and calibrated encoding masks are first processed by the initialization module, followed by an encoder–decoder backbone containing repeated spatiotemporal spectrum attention blocks to reconstruct the hyperspectral video. The Block progressively fuses multi-scale features through cascaded Former modules and a residual 1×1 convolution. Each

Former module combines three-dimensional convolution, self-attention, and feed-forward feature transformation. **(b)** Construction of the joint time-spectral mask. The pixelwise outer product between the temporal mask **Mt** and spectral mask **Mλ** produces the joint encoding tensor **Mc** with $T\times C$ channels. **(c)** The joint mask **Mc** and its channel-summed response **Ms** are combined with the measurement to generate the initialized measurement features. The mask and measurement features are then processed by dedicated three-dimensional convolutional modules before entering the encoder. **(d)** Comparison between DM-Net and an adapted EfficientSCI baseline. Because the original EfficientSCI accepts only a single temporal mask, the temporal and spectral masks were directly concatenated and mapped to the required $T\times C$ channels using a convolutional layer, while the remaining EfficientSCI architecture and reconstruction procedure were kept unchanged. Under the same backbone network and training protocol, DM-Net achieves 35.90 dB, compared with 30.79 dB for EfficientSCI and 35.61Db for time-spectral encoding.

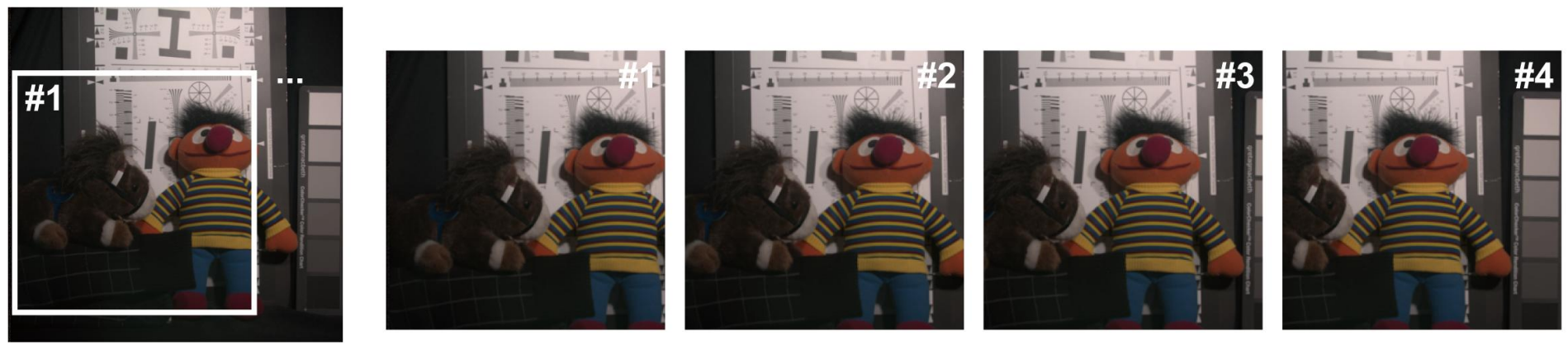

Supplementary Figure 5. Construction of synthetic dynamic hyperspectral training data. Representative examples of the synthetic dynamic hyperspectral sequences used for network training. Consecutive temporal frames are generated from static hyperspectral images by spatial cropping and reorganization, yielding multi-frame dynamic scenes with preserved spectral content and controlled temporal variation. These sequences are then used to simulate compressed measurements for supervised training of the reconstruction network.

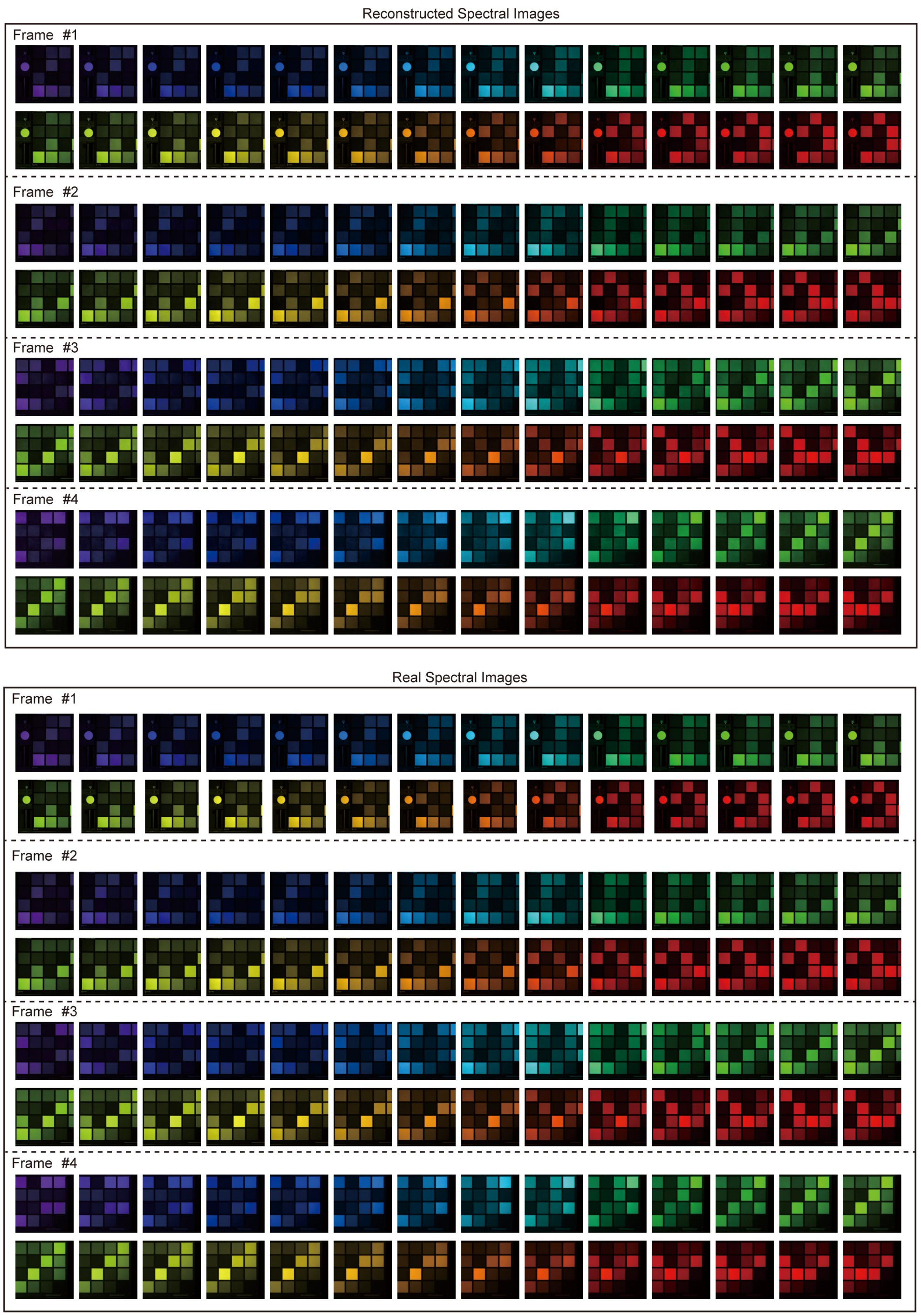

Supplementary Figure 6. Channel-wise hyperspectral reconstruction on synthetic dynamic data. Under a total output-to-readout dimensionality ratio

112:1, comprising a temporal ratio of 4:1 and a spectral ratio of 28:1, we test the proposed DM-NET. For each of the four recovered frames, the reconstructed 28-channel hyperspectral images are shown together with the corresponding ground-truth spectral images. **Fig. 2b** in the main text presents the synthesized pseudocolor visualization of one representative result, while this figure provides the complete channel-wise reconstruction. Additional representative examples and quantitative evaluation across the full test set are shown in **Supplementary Figure 7** and **Supplementary Table 1**, respectively.

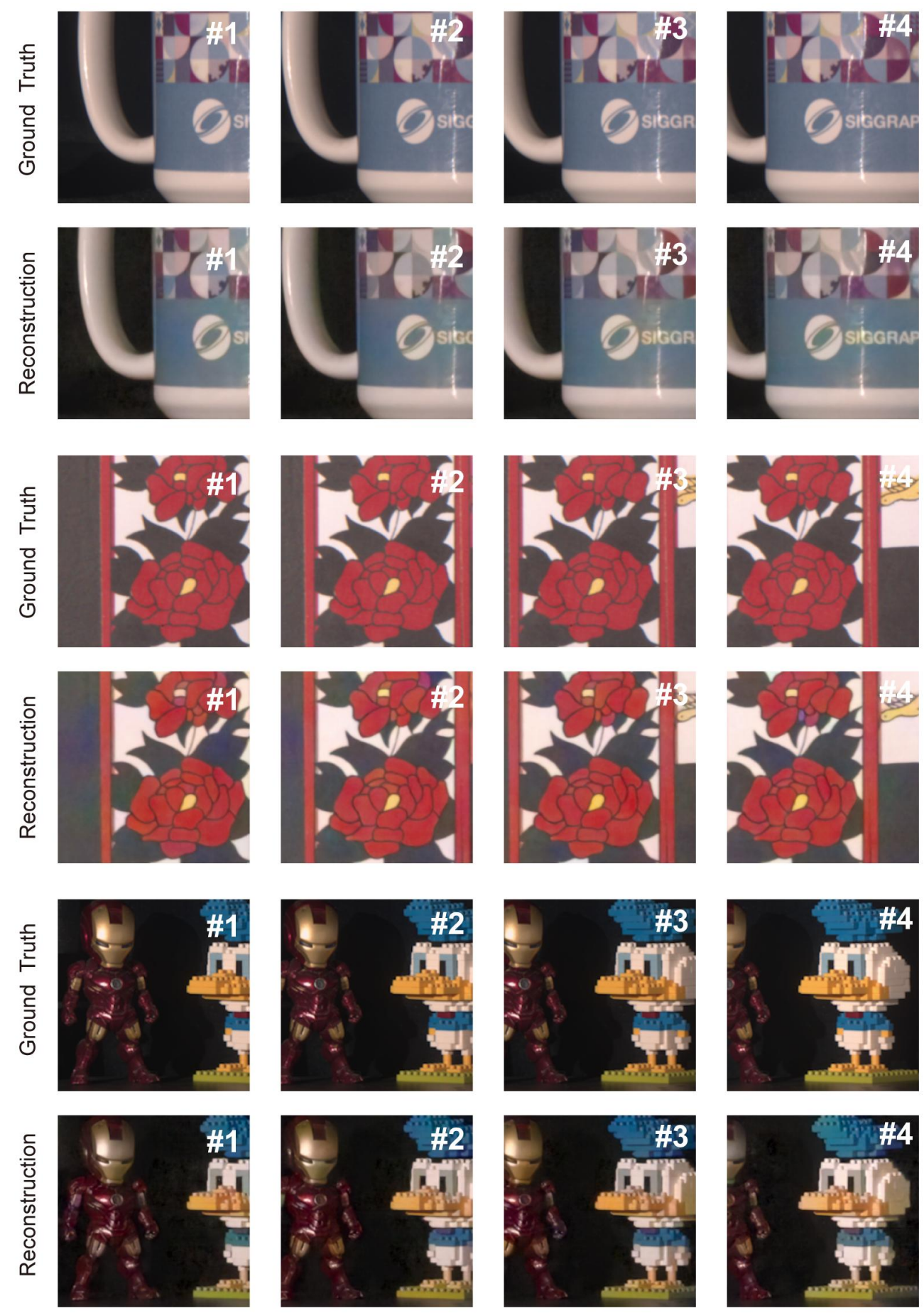

Supplementary Figure 7. Additional representative reconstruction results on synthetic dynamic hyperspectral data. Additional examples of synthesized pseudocolor reconstructions obtained under a nominal output-to-readout dimensionality ratio of 112:1. For each example, the four ground-truth frames are shown together with the corresponding reconstructed frames. The results demonstrate that the proposed network preserves the main spatial structures, temporal continuity, and color consistency across diverse dynamic scenes.

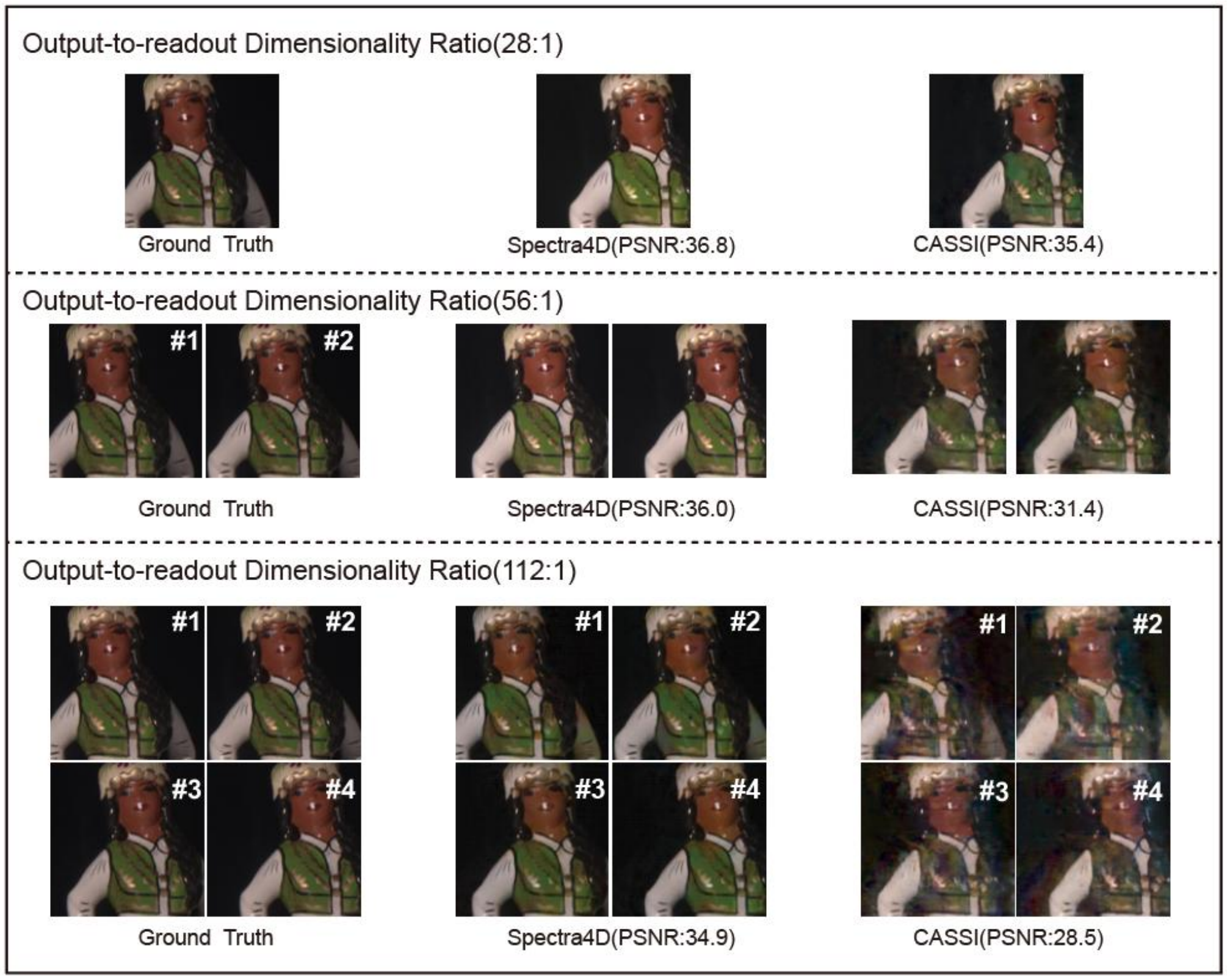

Supplementary Figure 8. Representative visual comparison between Spectra4D and temporally extended CASSI at different compression ratios. Representative synthesized pseudocolor reconstructions under three output-to-readout dimensionality ratios, 28:1, 56:1, and 112:1, corresponding to increasing temporal compression with the spectral ratio fixed at 28. For each setting, the ground-truth frames are compared with the reconstructions obtained by the proposed F-P based Spectra4D framework (F-P) and the temporally extended CASSI baseline. The displayed PSNR values indicate that Spectra4D consistently preserves higher visual fidelity across all compression settings, with the advantage becoming more pronounced under stronger compression. These representative results are consistent with the quantitative trend summarized in **Fig. 2e**.

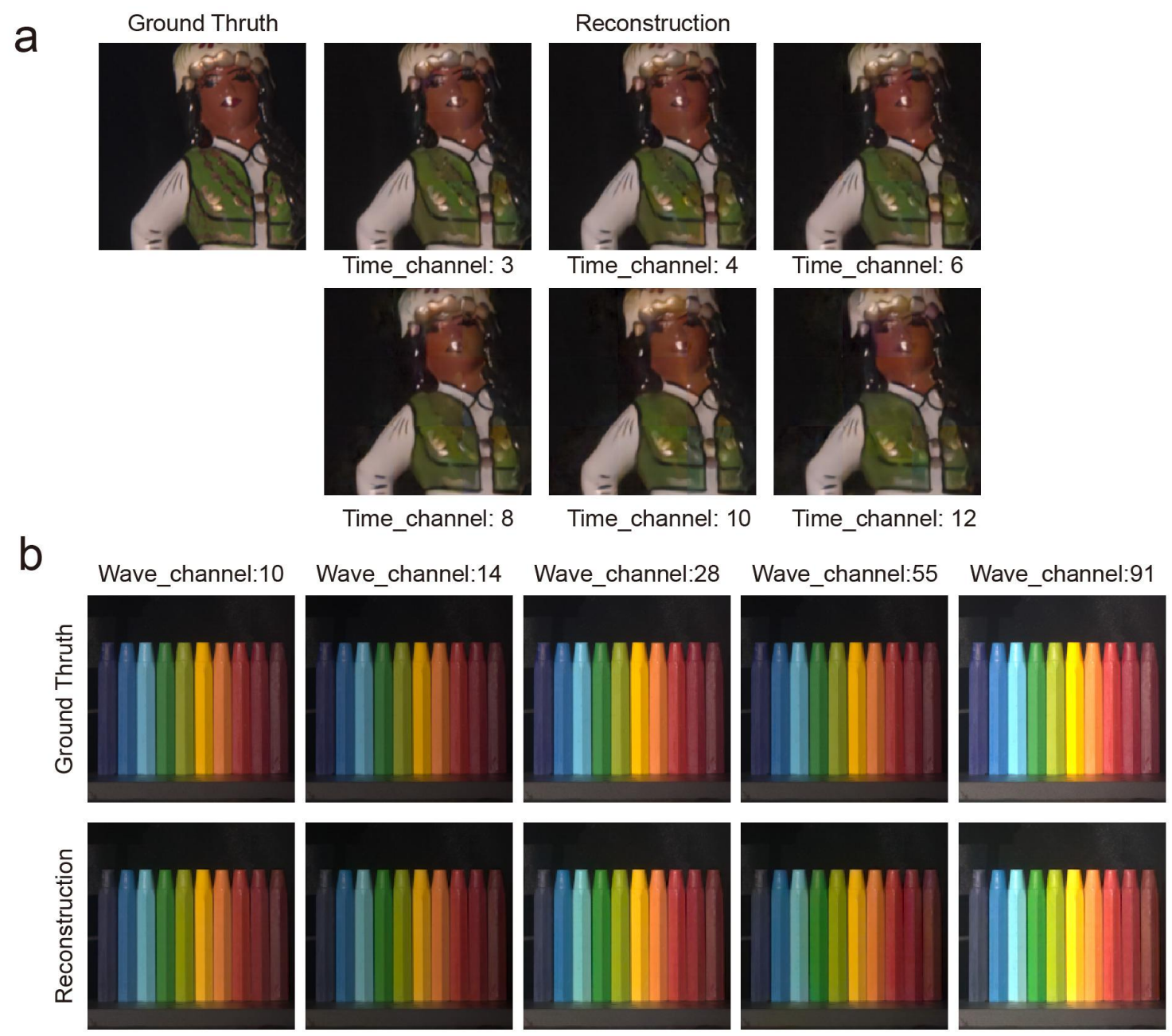

Supplementary Figure 9. Scalability of Spectra4D with increasing temporal and spectral output dimensions. The reconstruction complexity and GPU memory consumption are mainly determined by the total number of time-spectral output channels, T×C. Under the current RTX 3090 configuration, the maximum practical output dimension was approximately 120 channels. (a) Temporal scalability evaluated by fixing the spectral dimension at C=10 and increasing the temporal compression factor from T=3 to T=12. The reconstructed images retain the main spatial structures and textures as the temporal dimension increases. The corresponding quantitative results are summarized in Supplementary Table 3. (b) Spectral scalability evaluated without temporal compression (T=1) by increasing the spectral output dimension from C=10 to C=91. All configurations were independently trained and evaluated using the same forward model, datasets, noise conditions, and reconstruction protocol. The corresponding quantitative results are summarized in Supplementary Table 4.

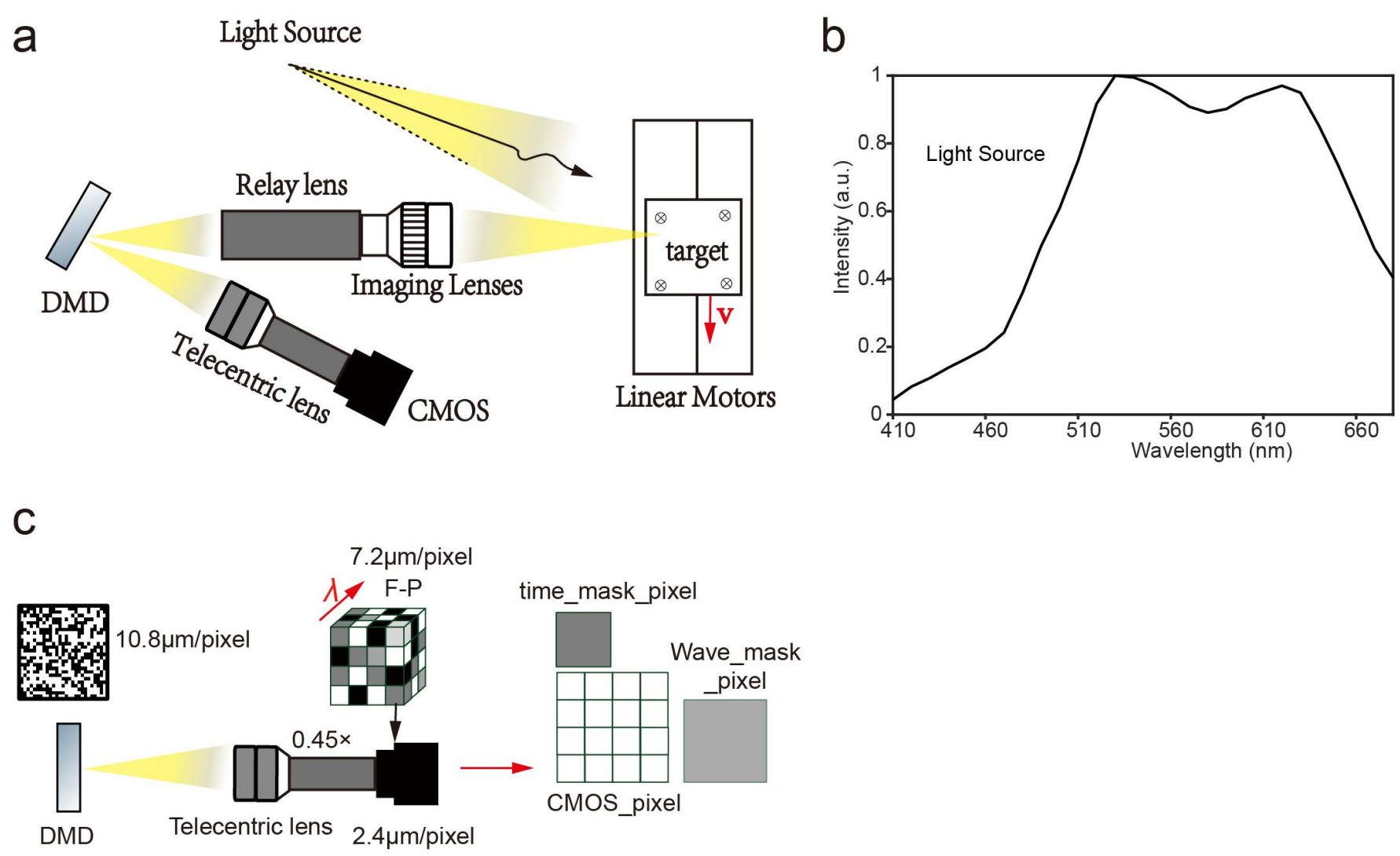

Supplementary Figure 10. Optical setup for quantitative evaluation and dynamic scene experiments. (a) Optical layout of the experimental platform used for the quantitative evaluation of Spectra4D and for the dynamic target reconstruction experiments. The target is mounted on a motorized linear stage to emulate controlled motion. Illumination from an external light source passes through the target and is imaged onto the DMD by the relay/imaging-lens system. After temporal modulation by the DMD, the encoded light is collected by a telecentric lens and recorded by the CMOS sensor. **(b)** Normalized spectrum of the light source used for the above experiments. Four consecutive measurements yielded a mean spectral fidelity of 99.984%, with intensity variations below 0.1581%. **(c)** Nominal encoding-scale mapping. The 10.8 μm DMD micromirror pitch is projected onto the CMOS plane with a magnification of 0.45×, corresponding to approximately 2×2 CMOS pixels with a 2.4 μm pixel pitch. Each 7.2 μm F–P cell nominally covers approximately 3×3 CMOS pixels. The temporal and spectral masks are calibrated directly on the CMOS pixel grid.

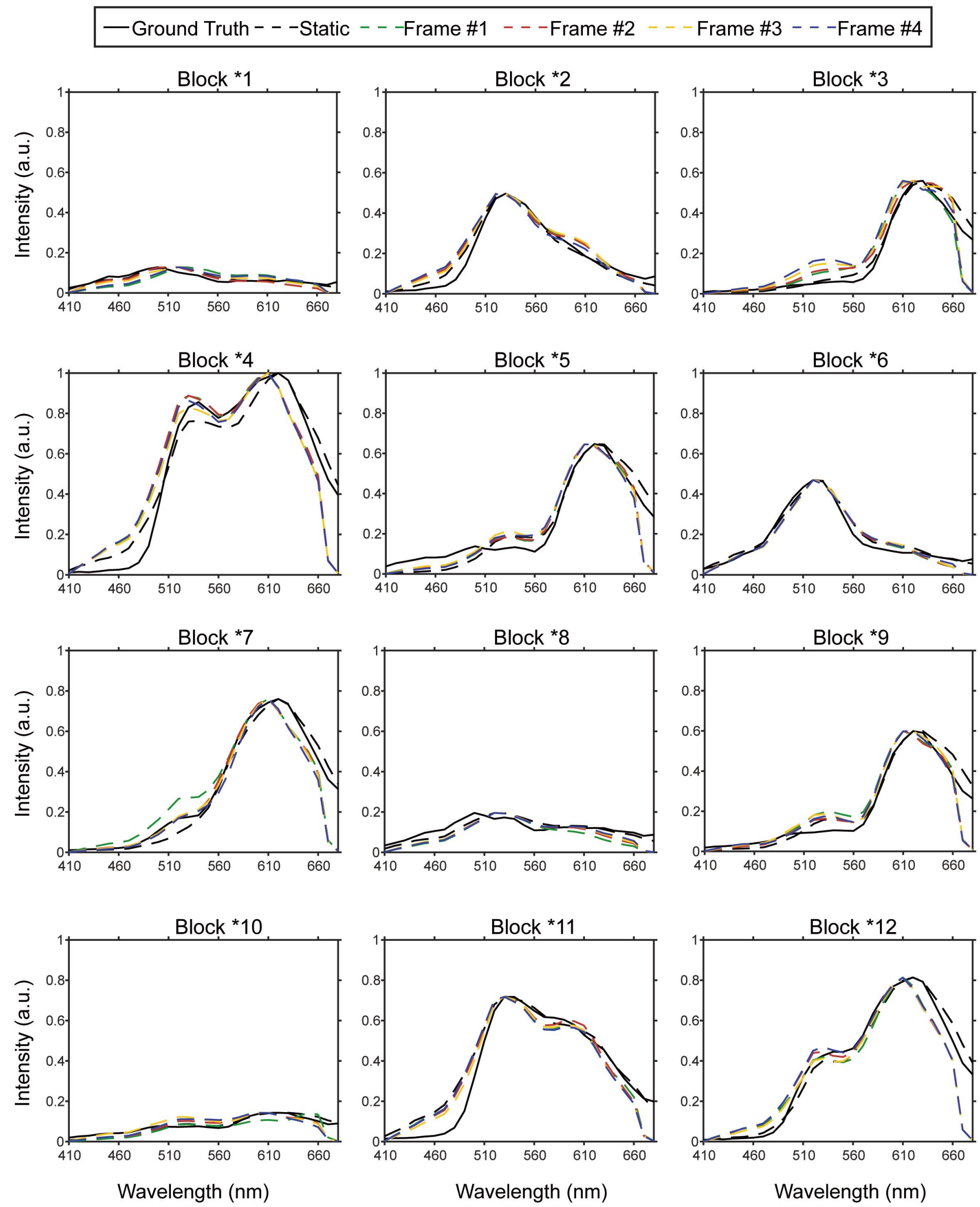

Supplementary Figure 11. Spectral comparison for all selected ColorChecker patches. Spectral curves for the 12 representative ColorChecker patches selected in **Fig. 3a**. For each patch, the spectrometer-measured ground truth is compared with the static hyperspectral reconstruction without temporal coding (Static) and the four temporally reconstructed frames (#1-#4). Overall, the reconstructed spectra preserve the main spectral profiles

and peak positions across different color patches, with deviations mainly appearing in low-intensity regions and at sharp spectral transitions.

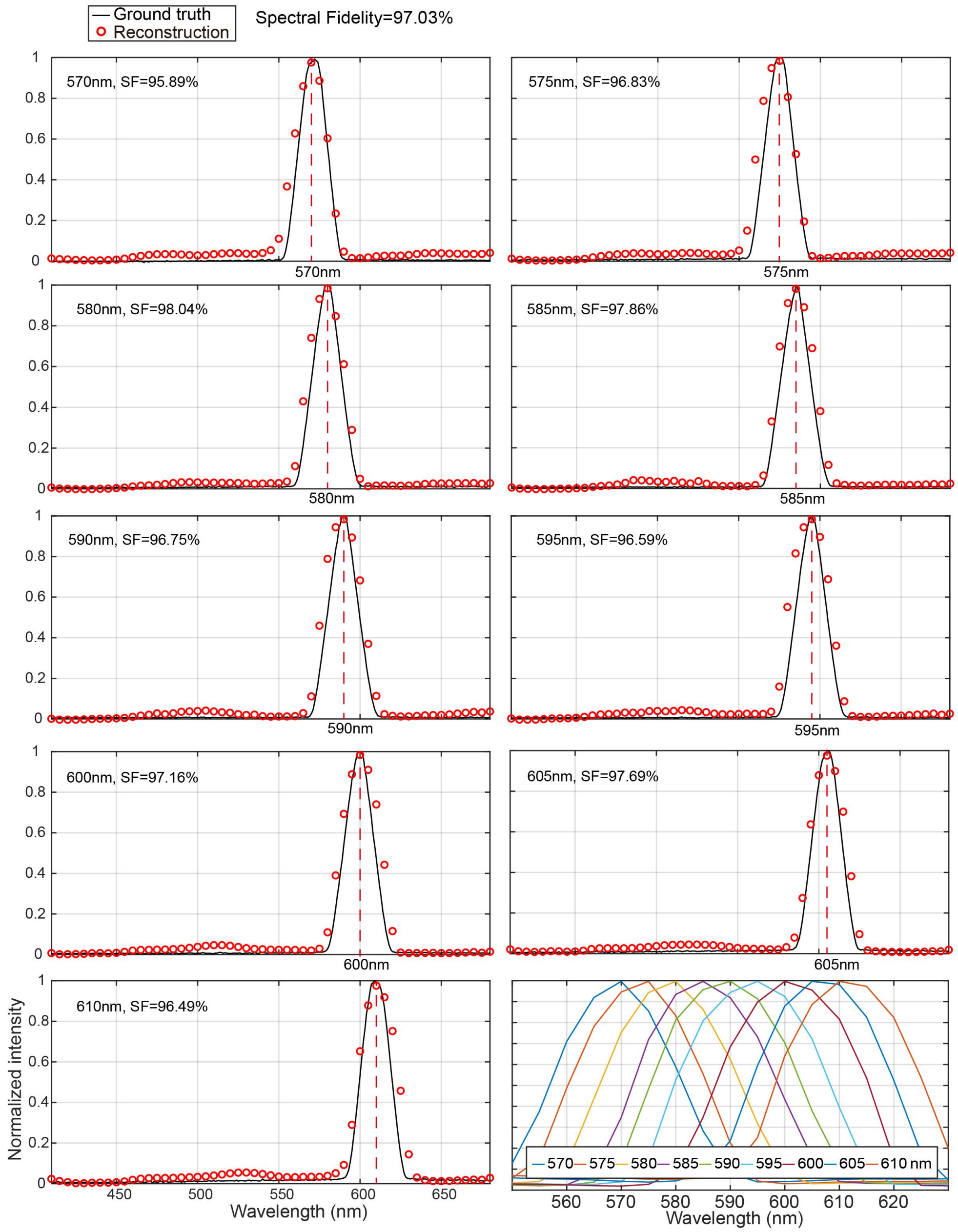

Supplementary Figure 12. Spectral-resolution experiment. Isolated spectral peaks were tuned from 570 to 610 nm in 5 nm center-wavelength steps. The reconstructed peaks accurately followed these wavelength shifts, with average spectral fidelities of 97.03%. The bottom-right panel overlays all reconstructed

spectral curves, showing that adjacent center-wavelength shifts of 5 nm remain clearly distinguishable. These results demonstrate an experimental spectral resolution sufficient to support the 10 nm channel spacing used for the 28 output channels over 410–680 nm.

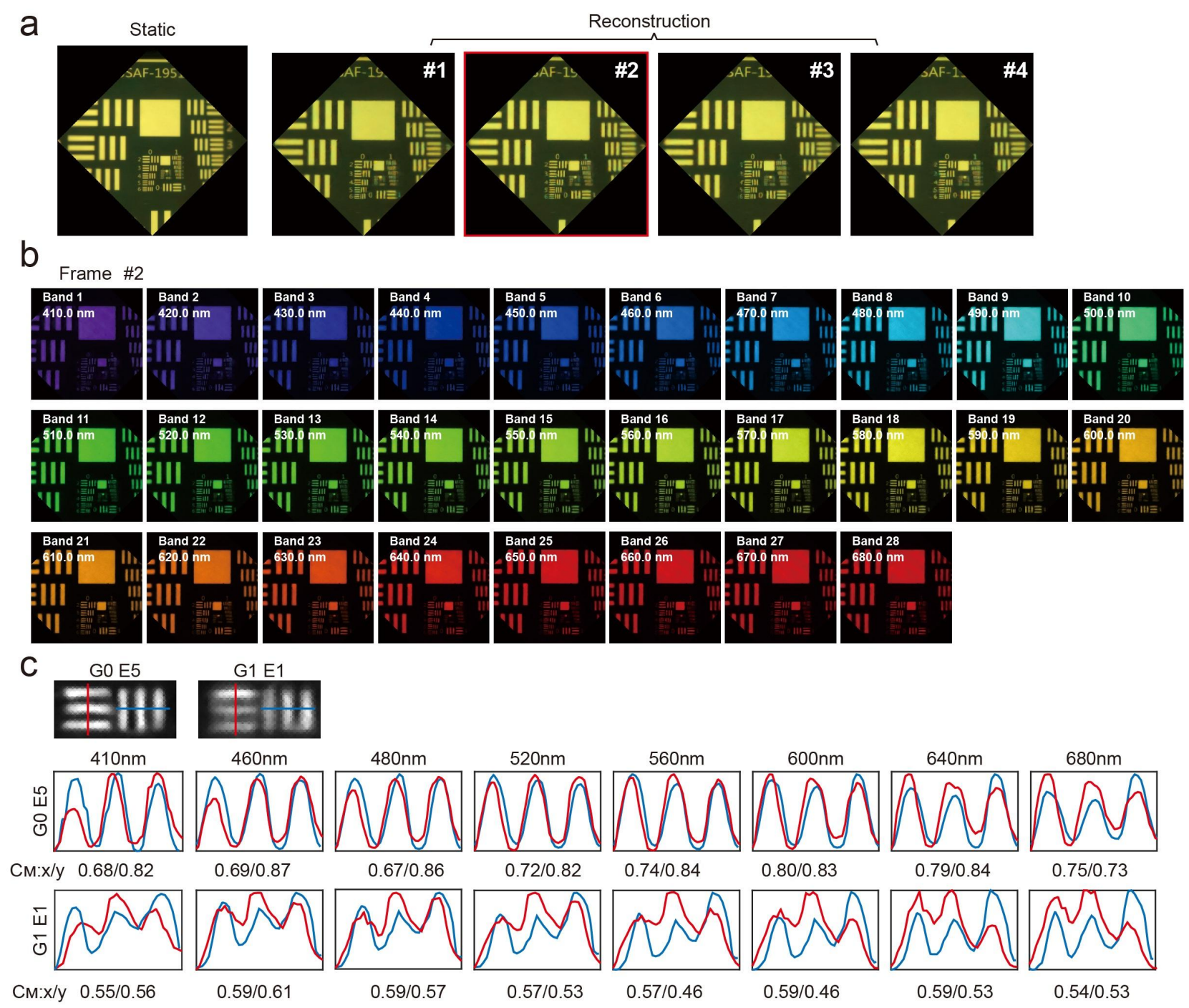

Supplementary Figure 13. Supplementary results for quantitative spatial-resolution evaluation with the USAF 1951 target. (a) Synthesized RGB image of the static hyperspectral reconstruction without temporal coding (Static) and the four temporally reconstructed frames recovered from a single compressed measurement (Reconstruction). **(b)** Channel-wise reconstructed hyperspectral images (410-680 nm) of the selected reconstructed frame (#2). The line-pair features of the USAF 1951 target remain clearly resolved across spectral channels, providing supplementary evidence that Spectra4D preserves fine spatial structures under four-fold temporal compression and supports the quantitative spatial-resolution analysis presented in **Fig. 3c-e**. **(c)** Intensity profiles and Michelson contrasts of Group 0 Element 5 (1.59 lp/mm) and Group 1 Element 1 (2 lp/mm) at representative wavelengths. The red and blue curves correspond to the two orthogonal sampling directions marked above. All measured contrasts remain above 0.46, demonstrating an effective spatial resolution of approximately 2 lp/mm across the reconstructed spectral range.

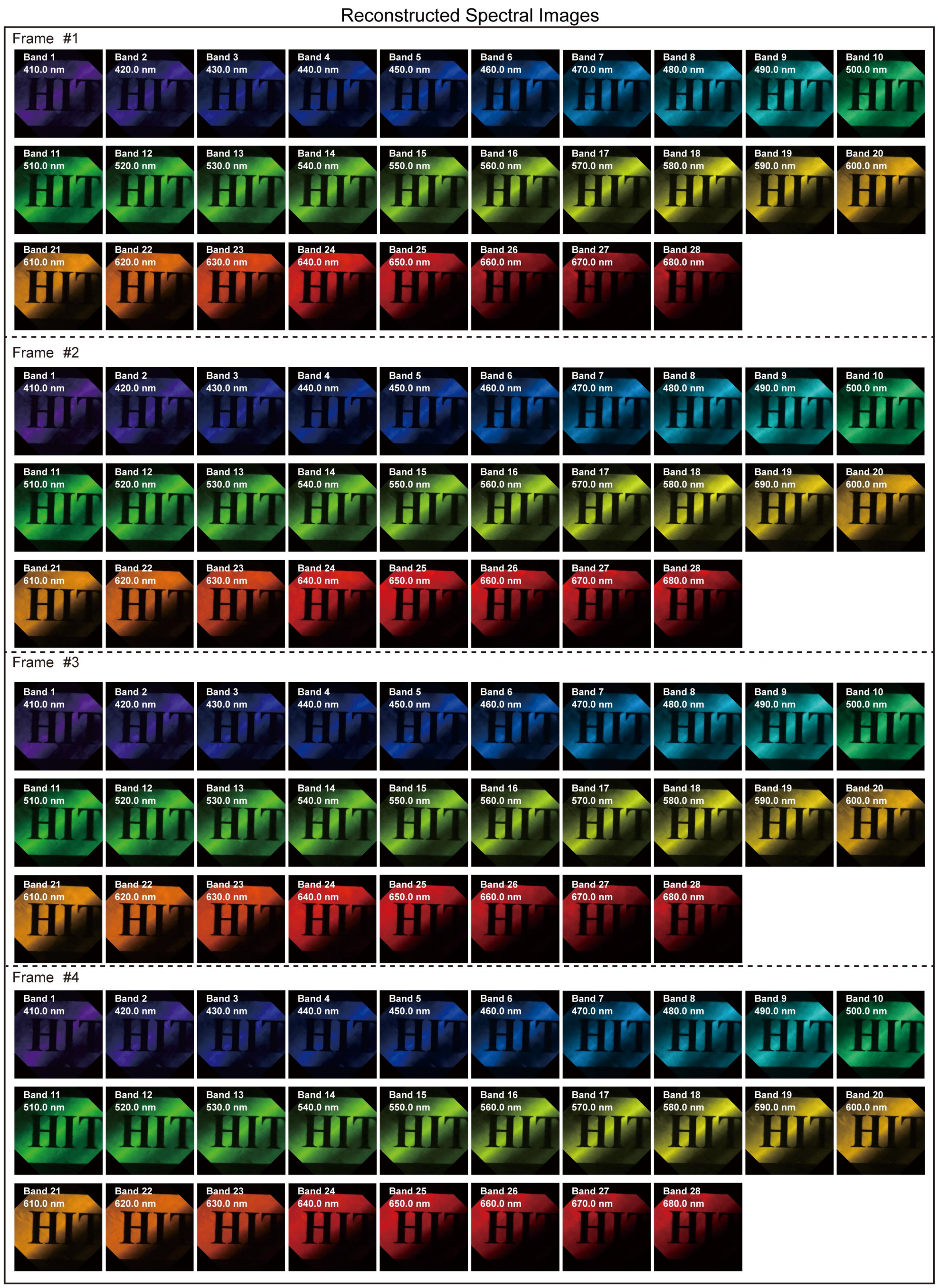

Supplementary Figure 14. Channel-wise reconstructed hyperspectral images of the planar dynamic target. Reconstructed hyperspectral images of the planar target for all 28 spectral bands (410 – 680 nm) across the four temporally recovered frames (#1-#4). The results show that the reconstructed

sequence preserves both the continuous spectral transition of the background and the sharp boundaries of the text features across all spectral channels and temporal frames, demonstrating stable recovery of spatiotemporal - spectral information under four-fold temporal compression.

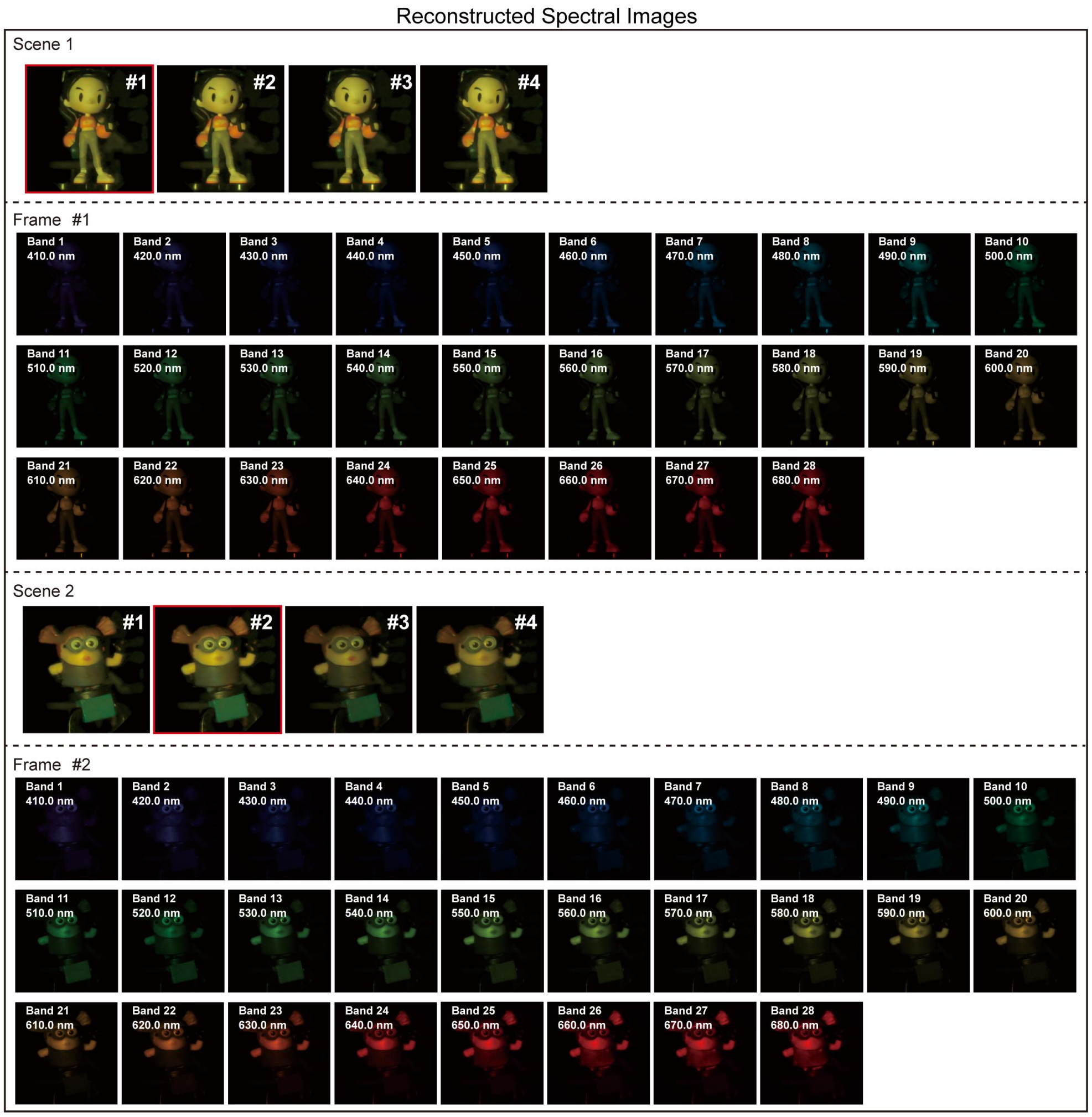

Supplementary Figure 15. Channel-wise reconstructed hyperspectral images of dynamic three-dimensional targets. Top: reconstructed RGB frames for Scene 1, together with the full 28-channel hyperspectral images (410-680 nm) of the highlighted frame (#1). Bottom: reconstructed RGB frames for Scene 2, together with the full 28-channel hyperspectral images of the highlighted frame (#2). The results show that Spectra4D preserves both inter-material spectral differences and fine spatial structures across dynamically varying three-dimensional scenes, including object contours, local textures, and wavelength-dependent reflectance variations.

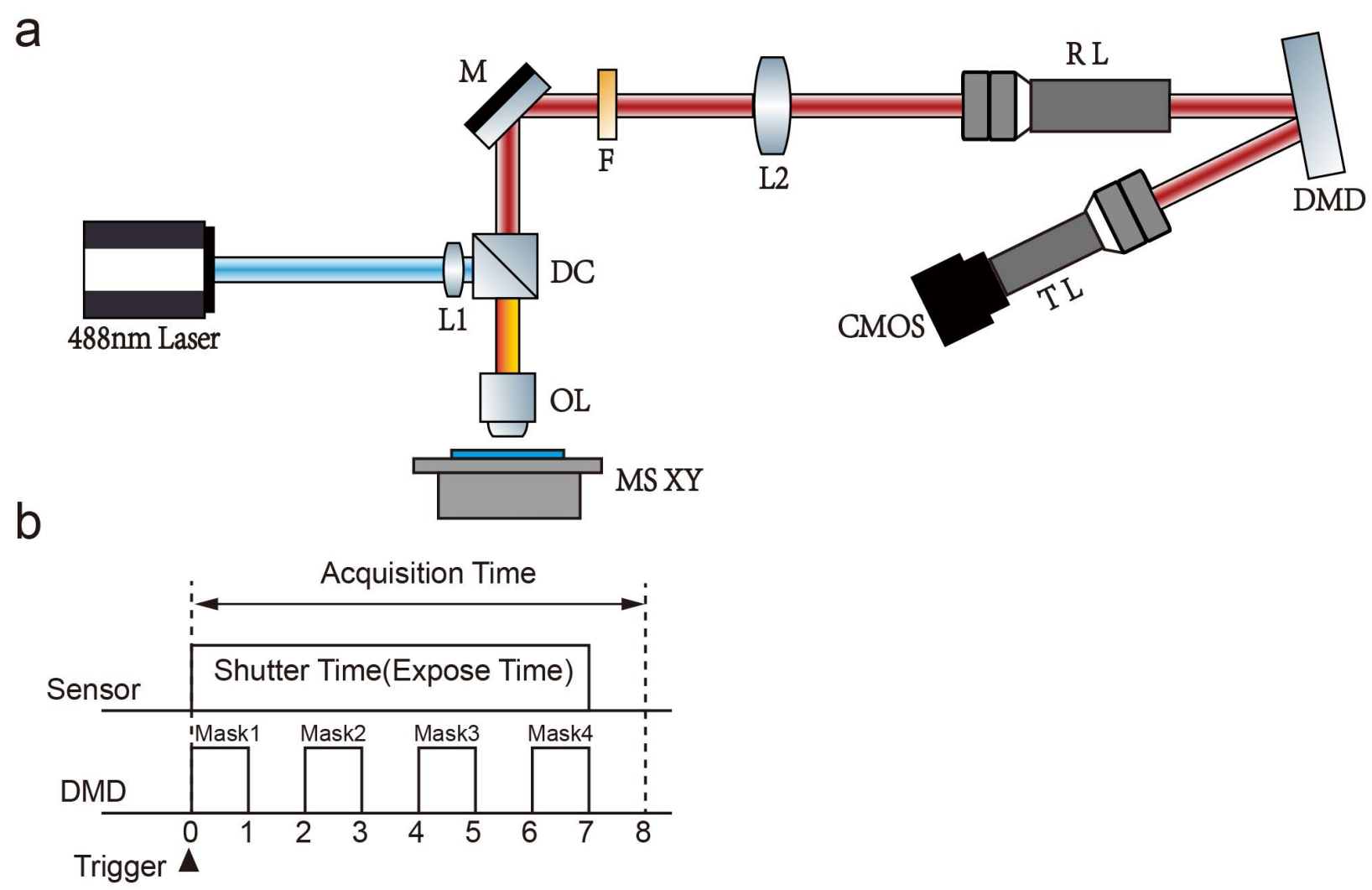

Supplementary Figure 16. Optical setup and synchronized acquisition timing for fluorescent microsphere tracking experiments. (a) A 488 nm laser is used for excitation. The fluorescence emitted from the sample is collected by the objective lens, separated by the dichroic mirror, filtered, and relayed to the DMD and CMOS camera for temporally encoded imaging. M: mirror; L: lens; DC: dichroic mirror; OL: objective lens; MSxy: xy microstage; F: Fluorescence filter; RL: Relay lens; TL: Telecentric lens; DMD: Digital Micromirror Devices. **(b)** Hardware-synchronized acquisition timing diagram at a camera frame rate of 50 fps. Four active DMD masks, each with a valid dwell time of 2.5 ms, are displayed during the 17.5 ms camera exposure, with a 2.5 ms all-off pattern inserted between adjacent masks. The final 2.5 ms all-off interval accommodates camera readout and data storage.

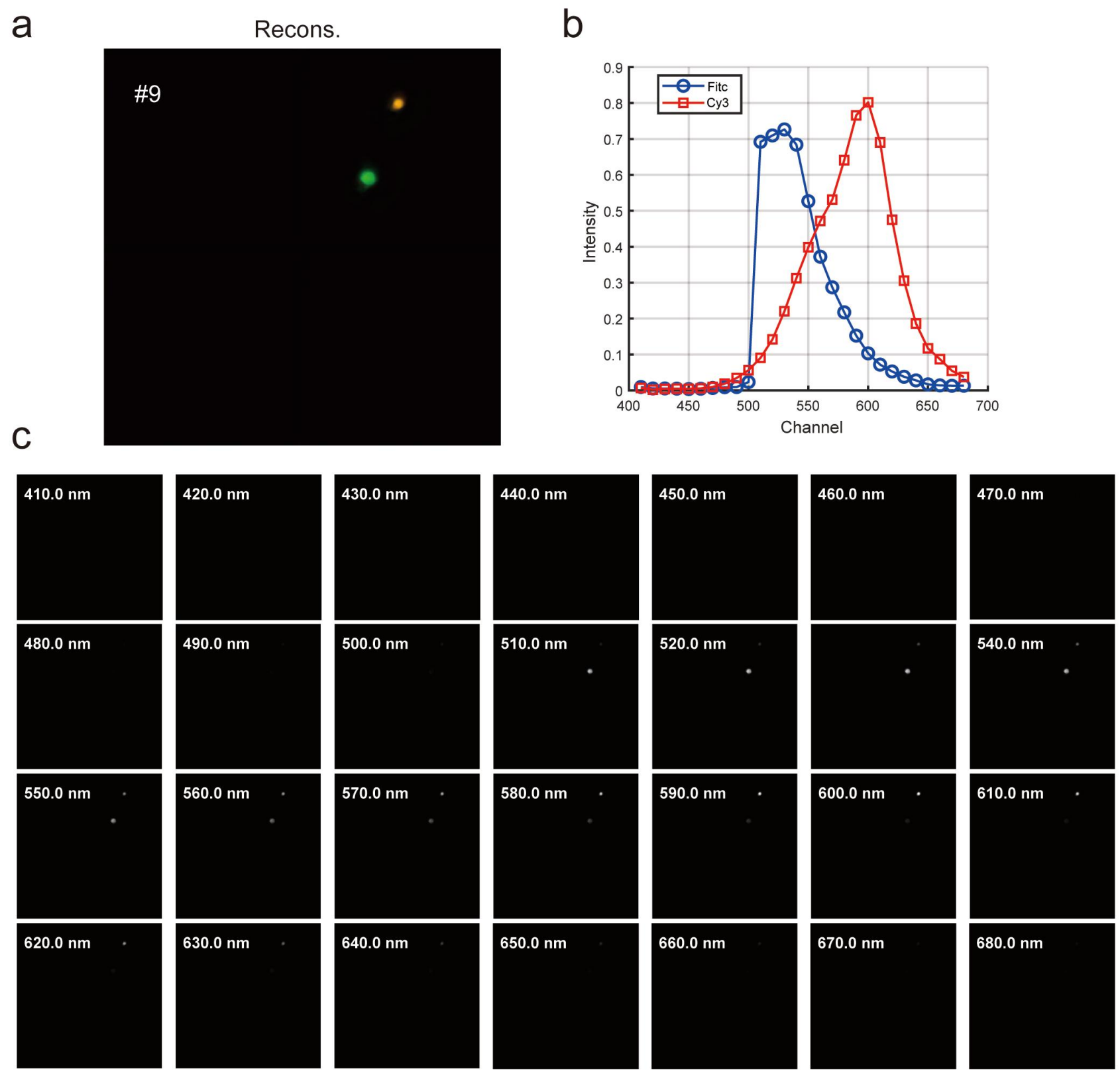

Supplementary Figure 17. Spectral discrimination of dual-colour fluorescent microspheres. (a) Synthesized RGB image reconstructed from the hyperspectral data cube, showing two fluorescent microspheres with distinct emission colors. **(b)** Reference emission spectra of the two microsphere species, corresponding to FITC and Cy3 fluorescence. **(c)** Channel-wise reconstructed hyperspectral images from 410 to 680 nm, showing the wavelength-dependent responses of the two microspheres. The FITC-labelled microsphere is dominant in the green spectral range, whereas the Cy3-labelled microsphere exhibits stronger emission in the orange-red spectral range.

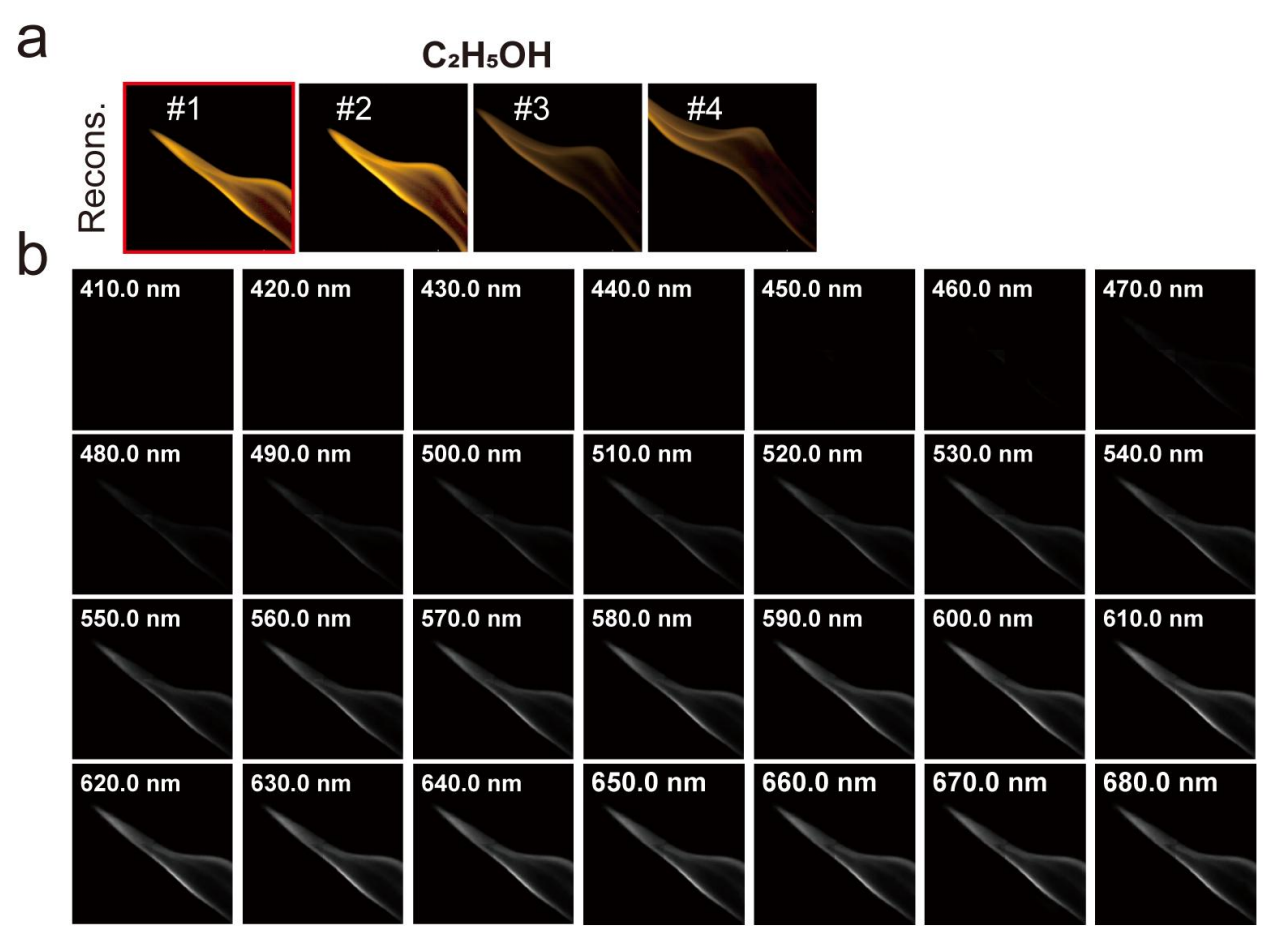

Supplementary Figure 18. Reconstructed hyperspectral frames and channel-wise images of the ethanol flame. (a) Synthesized RGB images of four reconstructed frames from the temporally compressed measurement of the ethanol flame. **(b)** Channel-wise reconstructed hyperspectral images from 410 to 680 nm for the highlighted frame (#1), showing the broadband spectral emission distribution of the undoped ethanol flame.

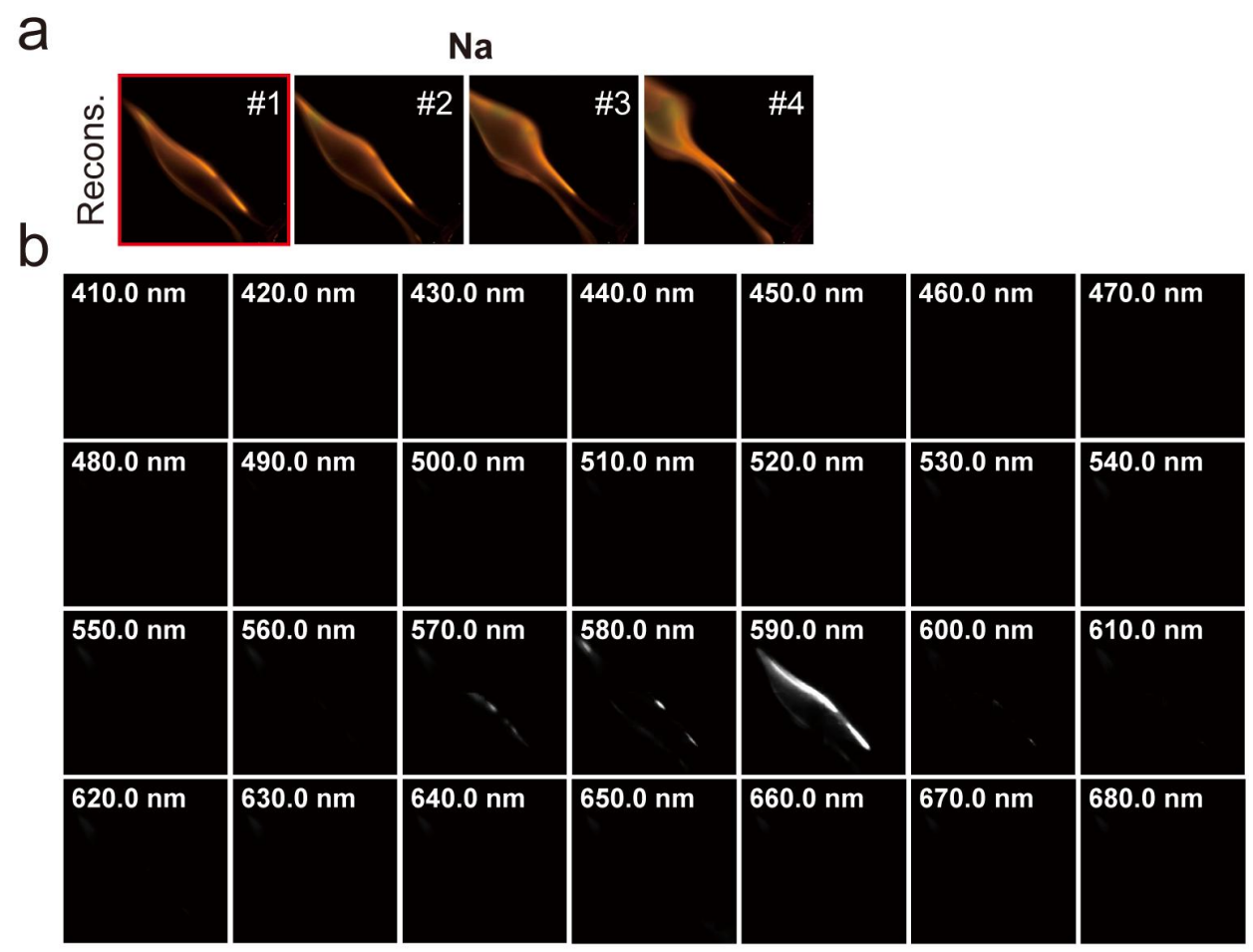

Supplementary Figure 19. Reconstructed hyperspectral frames and channel-wise images of the Na-doped ethanol flame. (a) Synthesized RGB images of four reconstructed frames from the temporally compressed measurement of the Na-doped flame. **(b)** Channel-wise reconstructed hyperspectral images from 410 to 680 nm for the highlighted frame (#1), showing the spectrally localized enhancement associated with sodium emission, particularly in the yellow spectral region.

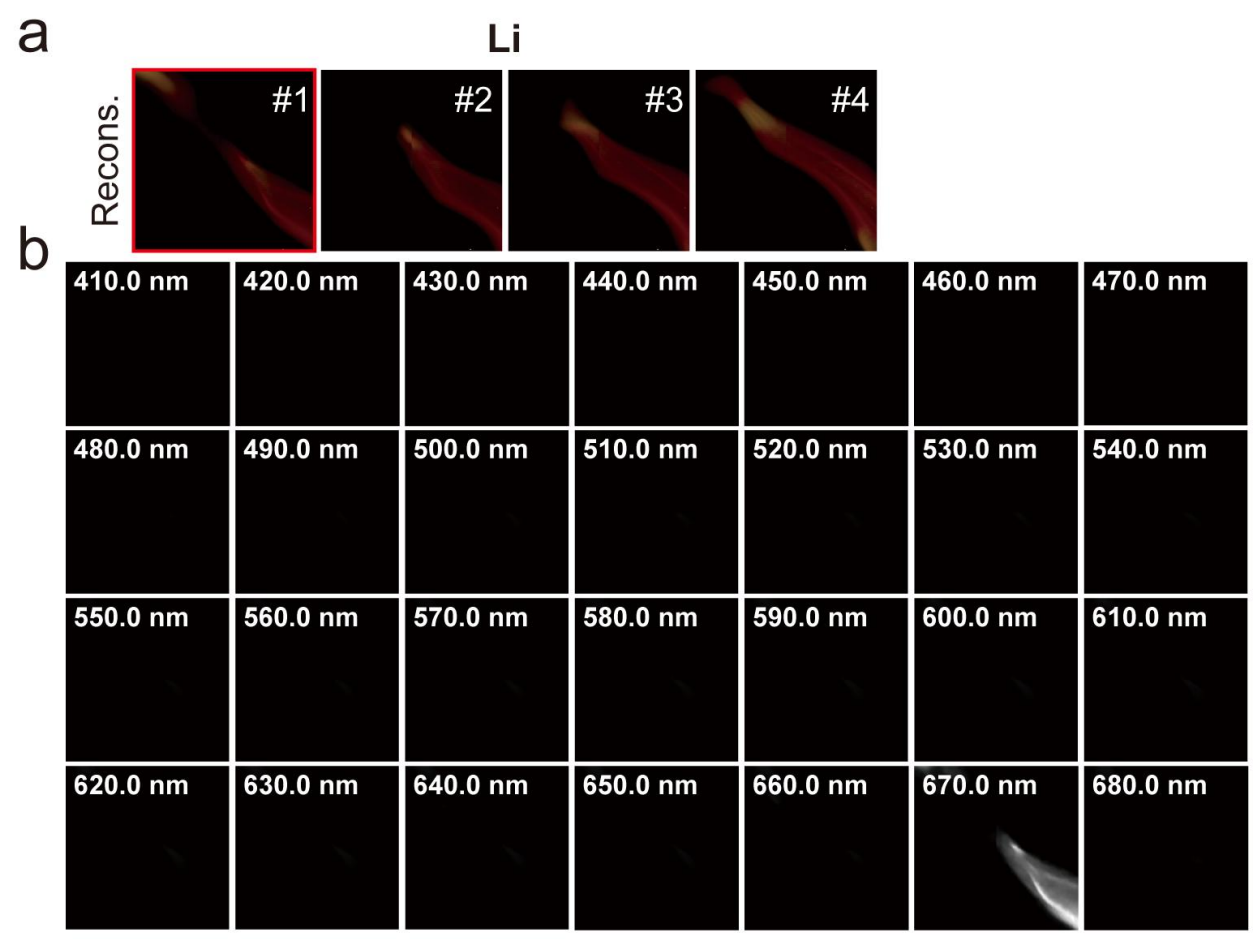

Supplementary Figure 20. Reconstructed hyperspectral frames and channel-wise images of the Li-doped ethanol flame. (a) Synthesized RGB images of four reconstructed frames from the temporally compressed measurement of the Li-doped flame. **(b)** Channel-wise reconstructed hyperspectral images from 410 to 680 nm for the highlighted frame (#1), showing the spectrally localized enhancement associated with lithium emission, particularly in the red spectral region.

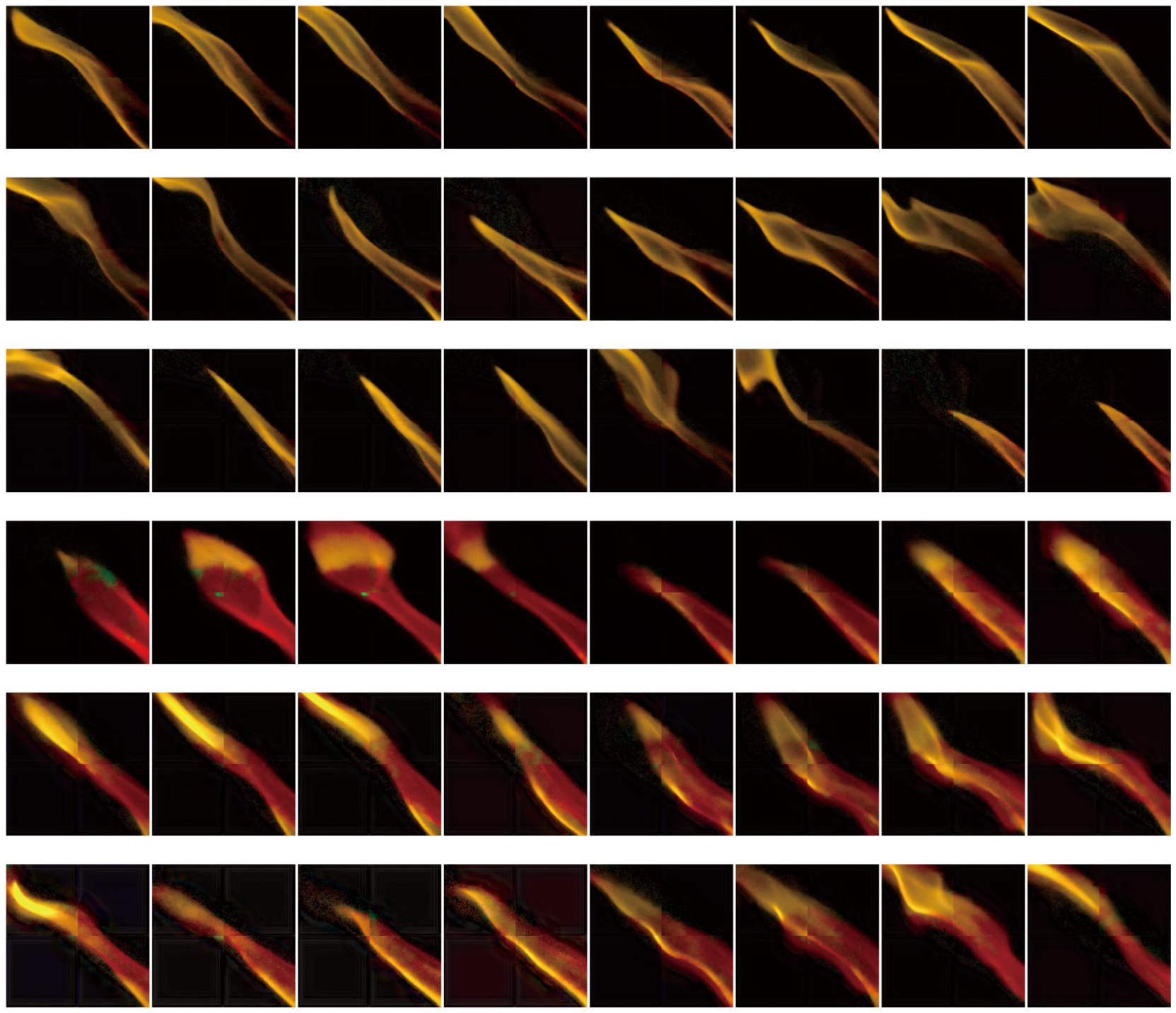

Supplementary Figure 21. Consecutive reconstructed RGB frames during lithium injection into the flame. Representative synthesized RGB images reconstructed from consecutive temporally compressed measurements acquired during lithium injection into the ethanol flame. The reconstructed frames capture the continuous spatiotemporal evolution of the flame after Li introduction, including the emergence and propagation of lithium-associated emission features and their coexistence with the background ethanol flame emission. These results further illustrate the ability of Spectra4D to resolve dynamic elemental composition changes and their spatial distribution during combustion.

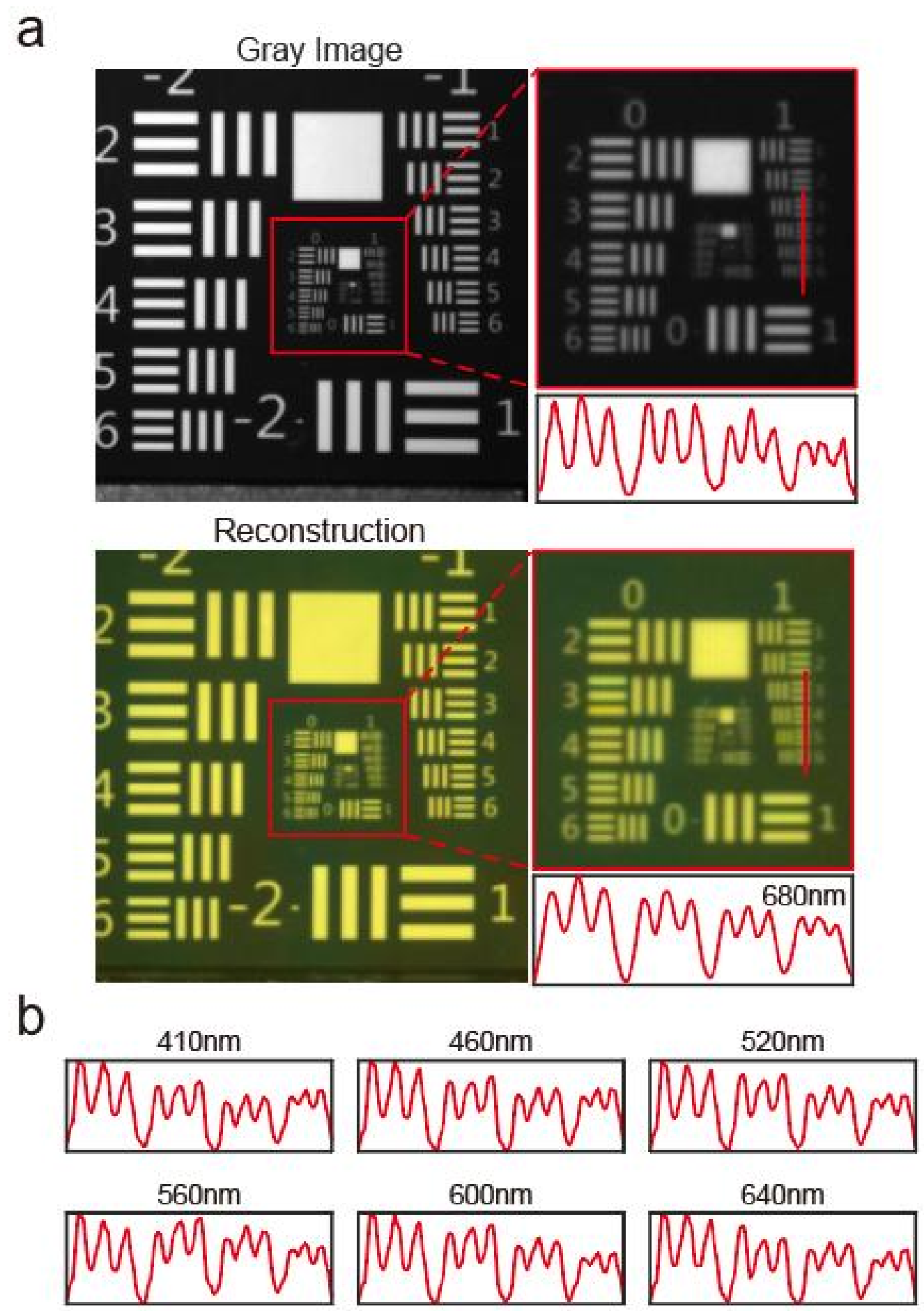

Supplementary Figure 22. Spatial-resolution comparison between bare-CMOS imaging and F–P-encoded reconstruction. (a) Bare-CMOS grayscale image and F–P-encoded reconstruction of the same stationary USAF 1951 target under identical imaging distance, lens, illumination, field of view, and exposure conditions. Temporal compression was disabled (T=1) to isolate the effects of spectral encoding and reconstruction. Both results resolve the same finest line-pair element, Group 1 Element 6. The yellowish reconstructed image reflects the spectrum of the halogen illumination. **(b)** Intensity profiles at representative wavelengths from 410 to 640 nm, demonstrating consistent preservation of spatial details across the reconstructed spectral channels.

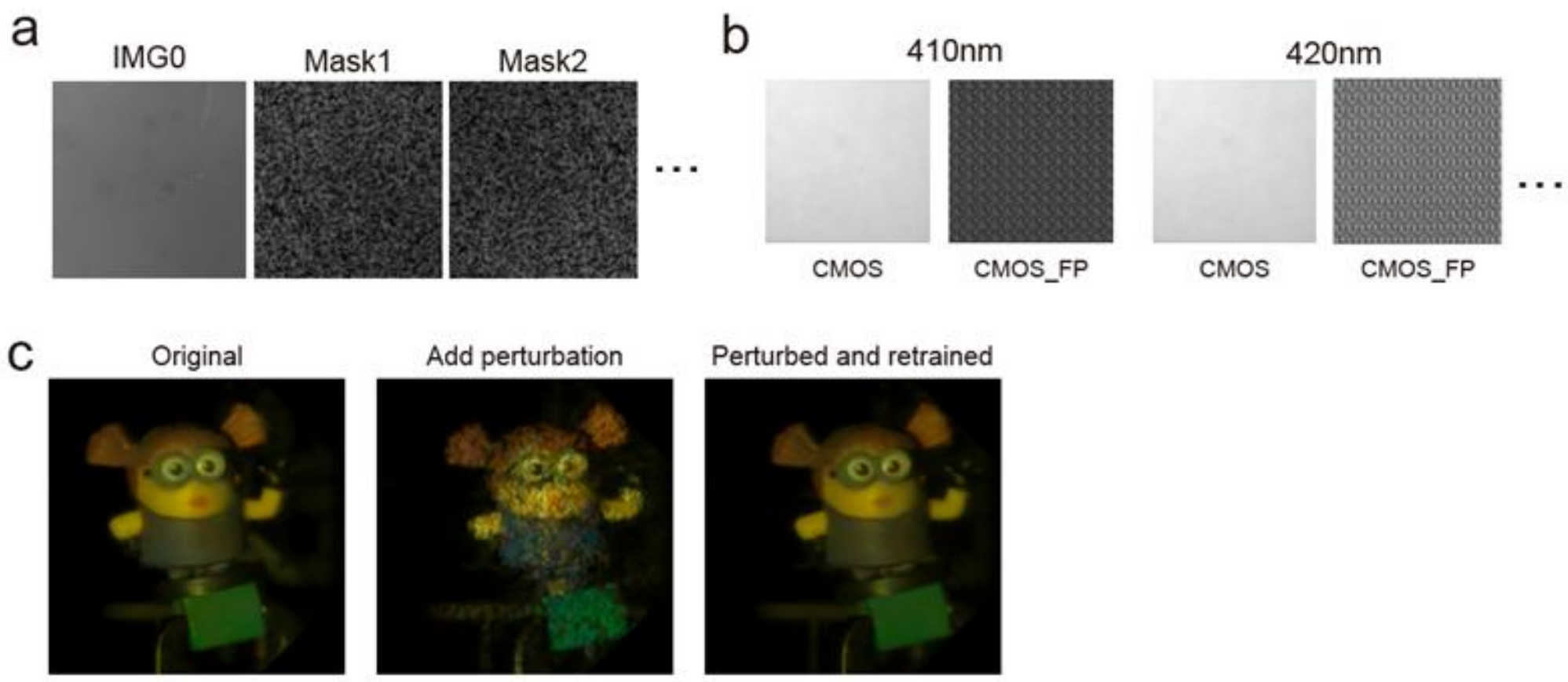

Supplementary Figure 23. Pixelwise calibration of temporal and spectral encoding masks. (a) Temporal-mask calibration. The all-on image and individual DMD-pattern images are recorded directly on the CMOS pixel grid and normalized to obtain the temporal masks. **(b)** Spectral-mask calibration. Images are acquired using matched CMOS sensors with and without the bonded F–P array under monochromatic illumination at each wavelength. Their pixelwise intensity ratios provide the calibrated F–P responses. **(c)** Reconstruction before and after mask perturbation. Applying an approximately 10 μm displacement in both spatial directions while retaining the original mask and model causes severe reconstruction artifacts. Recalibrating the perturbed mask and retraining from the original model weights restores the reconstruction, with a spectral fidelity exceeding 99.5% relative to the original result.

Supplementary Tables

Supplementary Table 1. Quantitative reconstruction performance on the synthetic test set. Trained and evaluated on synthetic dynamic hyperspectral sequences constructed from the CAVE dataset and the KAIST dataset using 300×300 patches.

Scene	PSNR	SSIM
1	37.0	0.97
2	36.9	0.97
3	36.2	0.96
4	43.3	0.98
5	34.1	0.97
6	35.8	0.97
7	36.4	0.96
8	32.0	0.95
9	33.9	0.96
10	33.1	0.96
11	36.7	0.98
Avg	35.9	0.97

Supplementary Table 2. Quantitative PSNR comparison among representative reconstruction methods using 256×256 patches.

	mst_s[1]	specat[2]	dauhst_5stg[3]	padut_3stg[4]	ours
PSNR:	21.82	26.85	28.02	28.92	34.95

Supplementary Table 3. Reconstruction performance at different temporal compression (T) factors with the spectral output fixed at 10 channels using 288×288 patches. (C=10)

T	3	4	6	8	10	12
PSNR	36.77	36.09	35.55	34.63	34.30	33.34

Supplementary Table 4. Reconstruction performance at different spectral output dimensions (C) without temporal compression and using 288×288 patches. (T=1)

C	10	14	28	55	91
PSNR	39.11	38.29	37.77	37.50	36.90

Supplementary Note

Supplementary Note 1: Network implementation and training details

The proposed DM-Net was implemented in PyTorch. The network takes the compressed measurement together with the calibrated spectral and temporal encoding masks as input, and reconstructs the corresponding multi-frame hyperspectral data cube. To improve the robustness and generalization ability of the network, data augmentation was applied during training. Specifically, each hyperspectral cube was randomly rotated by 0°, 90°, 180°, or 270°. This augmentation increases the diversity of spatial structures while preserving the underlying spectral information and temporal ordering.

The network was trained in a supervised manner using the mean squared error (MSE) between the reconstructed output and the ground-truth hyperspectral data cube. The loss function is defined as:

$$L = \frac{1}{N}\sum_{i} |x_i - \hat{x}_i|^2 \,, \tag{1}$$

where N is the number of training samples, x_i is the ground-truth hyperspectral data cube for the i-th sample, and $\hat{x}_i$ is the corresponding network output. All models were optimized using the Adam optimizer with β_1=0.9 and β_2=0.999. The initial learning rate was set to 0.0001, and training was performed for 900 epochs on four NVIDIA RTX 3090 GPUs.

Synthetic dynamic hyperspectral sequences were used as the main training data. For the flame-emission and fluorescent-microsphere reconstruction experiments, 30 independently acquired grayscale images were randomly assigned to individual wavelength channels between 410 and 680 nm to construct monochromatic hyperspectral samples. These samples accounted for

10% of the training data, while the remaining 90% comprised broadband CAVE samples.

The computational cost of DM-Net is reported. Using FP32 precision on an NVIDIA RTX 3090 GPU, DM-Net contains 8.824 million parameters, requires 508.38 ms for one inference, and has a peak GPU memory usage of 23.1 GB. The measured reconstruction throughput is 1.97 measurements per second and is primarily limited by GPU computation.

Supplementary Note 2: Spectral peak and size estimation of fluorescent microspheres

Spectral peak-center estimation of fluorescent microspheres.

For each tracked microsphere, a representative emission spectrum was extracted from the reconstructed hyperspectral cube by averaging all pixels within a circular region centered at the bead position. To estimate the emission peak with sub-channel precision, the local bead spectrum was matched to experimentally measured FITC and Cy3 reference templates rather than characterized by the discrete maximum of the 28-channel spectrum. Because these templates were acquired under the same experimental conditions, they preserved the actual spectral shape and linewidth of the fluorophores in the measurement environment. For each candidate template, a continuous wavelength shift was introduced and optimized together with amplitude scaling and baseline offset by least squares. The final peak-center wavelength was obtained from the intrinsic peak position of the matched template plus the optimal wavelength shift. This template-based strategy provided a robust estimate of the bead emission peak center under the actual imaging conditions and was used for subsequent population-level spectral analysis.

Radius estimation of fluorescent microspheres.

Bead radius was estimated from the reconstructed hyperspectral data using the spectrally summed intensity image. Local intensity maxima were first identified as bead candidates. Around each candidate, a local image patch was extracted and segmented using a relative intensity criterion, and only the connected component containing the local maximum was retained. This procedure isolated the effective bead image footprint while suppressing background fluctuations and interference from neighboring structures.

The bead radius was then calculated from the area of the retained region by converting it to an equivalent circular radius, yielding a stable image-domain estimate of bead size in each frame. The bead center was further refined using the intensity-weighted centroid of the segmented region to improve spatial consistency across frames. Finally, the radius was converted from pixels to micrometers using the calibrated effective object-plane pixel size. The resulting radius estimates were then used to quantify the bead-size distribution and its temporal stability throughout the reconstructed sequence.

Supplementary References

[1] Cai Y, Lin J, Hu X, et al. Mask-guided spectral-wise transformer for efficient hyperspectral image reconstruction[C]//2022 IEEE/CVF Conference on Computer Vision and Pattern Recognition (CVPR). IEEE, 2022: 17481-17490.

[2] Yao Z, Liu S, Yuan X, et al. Specat: Spatial-spectral cumulative-attention transformer for high-resolution hyperspectral image reconstruction[C]//2024 IEEE/CVF Conference on Computer Vision and Pattern Recognition (CVPR). IEEE, 2024: 25368-25377.

[3] Cai Y, Lin J, Wang H, et al. Degradation-aware unfolding half-shuffle transformer for spectral compressive imaging[J]. Advances in Neural Information Processing Systems, 2022, 35: 37749-37761.

[4] Li M, Fu Y, Liu J, et al. Pixel adaptive deep unfolding transformer for hyperspectral image reconstruction[C]//2023 IEEE/CVF International Conference on Computer Vision (ICCV). IEEE, 2023: 12913-12922.
